# The (Un)Conscious Mouse as a Model for Human Brain Functions: Key Principles of Anesthesia and Their Impact on Translational Neuroimaging

**DOI:** 10.3389/fnsys.2020.00008

**Published:** 2020-05-19

**Authors:** Henning M. Reimann, Thoralf Niendorf

**Affiliations:** ^1^Berlin Ultrahigh Field Facility (B.U.F.F.), Max-Delbrück Center for Molecular Medicine, Helmholtz Association of German Research Centers (HZ), Berlin, Germany; ^2^Experimental and Clinical Research Center, A Joint Cooperation Between the Charité Medical Faculty and the Max-Delbrück Center for Molecular Medicine, Berlin, Germany

**Keywords:** mouse, fMRI–functional magnetic resonance imaging, anesthesia, translational mouse-human, brain functional connectivity, EEG, Ca^2+^ imaging, MIND signature

## Abstract

In recent years, technical and procedural advances have brought functional magnetic resonance imaging (fMRI) to the field of murine neuroscience. Due to its unique capacity to measure functional activity non-invasively, across the entire brain, fMRI allows for the direct comparison of large-scale murine and human brain functions. This opens an avenue for bidirectional translational strategies to address fundamental questions ranging from neurological disorders to the nature of consciousness. The key challenges of murine fMRI are: (1) to generate and maintain functional brain states that approximate those of calm and relaxed human volunteers, while (2) preserving neurovascular coupling and physiological baseline conditions. Low-dose anesthetic protocols are commonly applied in murine functional brain studies to prevent stress and facilitate a calm and relaxed condition among animals. Yet, current mono-anesthesia has been shown to impair neural transmission and hemodynamic integrity. By linking the current state of murine electrophysiology, Ca^2+^ imaging and fMRI of anesthetic effects to findings from human studies, this systematic review proposes general principles to design, apply and monitor anesthetic protocols in a more sophisticated way. The further development of balanced multimodal anesthesia, combining two or more drugs with complementary modes of action helps to shape and maintain specific brain states and relevant aspects of murine physiology. Functional connectivity and its dynamic repertoire as assessed by fMRI can be used to make inferences about cortical states and provide additional information about whole-brain functional dynamics. Based on this, a simple and comprehensive functional neurosignature pattern can be determined for use in defining brain states and anesthetic depth in rest and in response to stimuli. Such a signature can be evaluated and shared between labs to indicate the brain state of a mouse during experiments, an important step toward translating findings across species.

## Introduction

Much of our understanding of human brain functions comes from murine studies. The ease of genetic modification and other practical and financial issues have made the mouse the best-explored mammalian model organism in neuroscience. A multitude of murine protocols and repositories provide reliable benchmarks in today’s brain research (including the *Allen Brain Project*^1^, the *Blue Brain Project*^2^, and the *Mouse ENCODE Project*^3^; Lein et al., 2007; Sunkin et al., 2013; Erö et al., 2018; Keller et al., 2018; Frankish et al., 2019). Still, it remains an open question to what extent, and under what conditions, findings from the mouse can be translated into an understanding of human brain functions. Although the mouse brain is not merely a miniaturized version of the human brain, its comparably small and flat neocortex exhibits striking functional and structural similarities, and the subcortical architecture is evolutionarily largely preserved (Ventura-Antunes et al., 2013; Hofman, 2014; Glickfeld and Olsen, 2017; Halley and Krubitzer, 2019).

In recent years, technical and procedural advances have brought functional magnetic resonance imaging (fMRI) to the field of murine neuroscience. The unique capacity of fMRI to measure functional brain activity non-invasively and across the entire brain relies on tight neurovascular coupling, in which increased neural activity triggers local elevations in cerebral blood flow (CBF), cerebral blood volume (CBV), and blood oxygenation (Hamilton et al., 2010; Hall et al., 2014). Each of these hemodynamic parameters can be assessed by fMRI as a surrogate for neural activity. Blood oxygenation level-dependent (BOLD) fMRI is the most popular approach due to its high sensitivity and comparably fast acquisition times (Ogawa et al., 1990; Kim, 2018). Modern MR scanners operating at ultrahigh magnetic field strengths (≥7 T) have been tailored for use with small rodents, and can achieve a relative spatial resolution analogous to that commonly used in human fMRI (voxels per anatomical region; voxel size of ~200 μm^3^). This permits direct comparisons of large-scale murine and human brain functions, and opens up opportunities to use a plethora of genetically engineered models to clarify a wide range of clinical and basic neuroscience issues—from the pathogenesis of neurological disorders to fundamental questions about consciousness.

Although the first report on murine fMRI was published more than two decades ago (Huang et al., 1996), its application to mice continues to require extensive refinements (Mandino et al., 2020). Aside from technical issues including signal amplification from the small mouse brain or its vulnerability to physiological perturbations, a fundamental problem of interspecies translation involves the *non-voluntary task conundrum*. In comparison to (adult) humans, a mouse has no interest in participating in an fMRI study. Head fixation, body restraint, and habituation to the acoustic noise produced by the MR gradients (up to 115 dB sound pressure) cause enormous stress to this remarkably rousable creature, which is far less amenable to training than rats (Jonckers et al., 2014, 2015; Low et al., 2016b; Dopfel and Zhang, 2018). Studying unbiased nociception in mice is almost impossible; even the application of aversive stimuli, like mild cooling of the paw, causes immediate withdrawal, and functional patterns across the brain reflect not only stress, fear, and anticipation, but also unrelated motor and sensory responses. This introduces tremendous complexity to fMRI of the awake mouse.

Low-dose anesthetic protocols are commonly applied in mouse fMRI to address these problems and to alleviate potential suffering due to stress, fear, and pain. The goal is to achieve high-quality translational data from sedated, i.e., calm, relaxed, and undistracted subjects. However, anesthetics have been shown to impair neural transmission (Baumgart et al., 2015; Hemmings et al., 2019) and also affect other aspects of murine physiology including hemodynamics (Franceschini et al., 2010; Masamoto and Kanno, 2012), thermoregulation (Reimann et al., 2016), respiration (van Alst et al., 2019), and cardiovascular control (Sinclair, 2003; Low et al., 2016a) in a dose-dependent manner. All these parameters can affect neurovascular coupling, which links the BOLD effect to the activity of neural populations. Therefore, anesthetic protocols in fMRI face two major challenges: first, to preserve functional *brain states* based on neural oscillation and transmission characteristics (see “Brain States, Anesthetic Depth, and Murine Consciousness” section), and second, to maintain hemodynamic integrity (see “Murine fMRI and Hemodynamic Integrity” section).

Recognizing the progress, opportunities, and challenges of murine functional brain mapping, this work provides a review of the literature on current anesthetic protocols and their meaning for murine functional neuroimaging. The aim is to introduce the basic principles of anesthesia to better understand and interpret the outcome of murine fMRI studies, and to develop novel anesthetic protocols and monitoring strategies dedicated to promoting reproducible and translational neuroimaging.

## Brain States, Anesthetic Depth, and Murine Consciousness

In the murine as in the human brain, neural information is processed against a background of spontaneous, ongoing activity generated by the promiscuous firing of neurons or structured patterns of neural populations. These oscillatory dynamics can be assessed *via* invasive and non-invasive neural readout techniques, such as electroencephalography (EEG), local field potentials (LFP), or calcium (Ca^2+^) imaging, yielding distinct spatiotemporal profiles that can be considered as signatures of distinct brain states.

### Cortical States of Wakefulness

**Figure 1:**
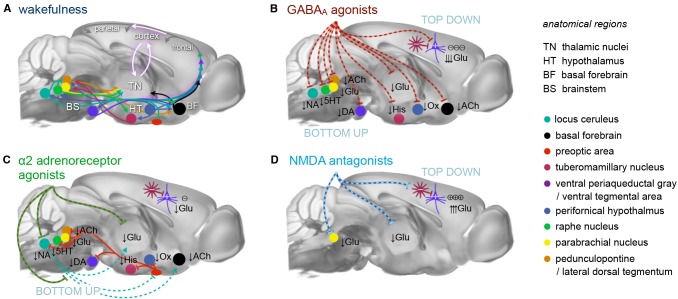
Principal mechanisms of anesthesia.** (A)** Brain states are governed by a highly interconnected assembly of subcortical (arousal) nuclei. These nuclei employ distinct transmitter systems, including glutamate (Glu), noradrenaline (NA), serotonin (5HT), dopamine (DA), acetylcholine (ACh), histamine (His), and orexin (Ox). They project to the cortex directly and *via* higher-order nuclei of the thalamus. Thalamus and cortex are densely interconnected and heavily exchange information. Synchronous rhythmic thalamocortical activity can set the phase relations of distant cortical areas. Similar phase relations facilitate information transfer across the cortex and from frontal to parietal regions. All anesthetics generate distinct thalamocortical rhythms and alter the phase relationship of transmitting and receiving areas, leading to a successive breakdown of cortico-(thalamo-)cortical communication and eventually loss of consciousness. **(B)** GABA_A_ agonists, including volatile ethers, affect brain states both top-down and bottom-up by inhibiting excitatory neurons in subcortical nuclei and directly in the cortex. **(C)** α2AR agonists exert their effects bottom-up mainly by inhibiting the locus coeruleus, which leads to a disinhibition of sleep promoting neurons in the preoptic area of the hypothalamus. **(D)** *N*-methyl-d-aspartate (NMDA) antagonists act primarily top-down in a dual mode: at low doses by inhibiting inhibitory interneurons leading to cortical excitation, and at higher dosages by also inhibiting excitatory cortical pyramidal neurons. Further suppression of nociception and arousal is mediated by blocking the parabrachial nucleus. These three routes present the major principles of anesthesia. All anesthetics in current use in preclinical fMRI act in one of these ways. Inspired by Franks (2008), Lee and Dan (2012) and Akeju and Brown (2017), plotted on an MR reference template of the Allen mouse brain atlas (Bakker et al., 2015).

Awake brain states undergo constant alterations in response to changing contexts of arousal, attention, and behavior (Steriade, 2000; Pfaff, 2005; Olcese et al., 2018; Poulet and Crochet, 2019). The spectrum of EEG patterns during these states ranges from synchronous low-frequency oscillations (<8 Hz) during quiet wakefulness to structured higher-frequency oscillations (~8–100 Hz) during attentional tasks and highly desynchronized high-frequency oscillations during aroused states (Petersen et al., 2003; Palva et al., 2005; Fries et al., 2007; Sachidhanandam et al., 2013; McGinley et al., 2015a; Olcese et al., 2018; Poulet and Crochet, 2019). The level of *cortical activation* (i.e., higher-frequency oscillations) is positively correlated to the level of general arousal, which is mediated by modulatory input from subcortical cholinergic and monoaminergic (dopaminergic, noradrenergic, serotonergic, and histaminergic) nuclei (Figure 1A; Aston-Jones et al., 1999; Pfaff, 2005; Harris and Thiele, 2011; Lee and Dan, 2012; McGinley et al., 2015b; Lecrux and Hamel, 2016; Ma et al., 2018). These arousal nuclei project into the cortex either directly or *via* the medial thalamus, and govern cortical states at both the global and local levels. Defined cortical regions have been shown to be remotely modulated, for instance, by inhibitory projections of the thalamic reticular nucleus (Lewis et al., 2015; Herrera et al., 2016; Fernandez et al., 2017) or excitatory projections of cholinergic neurons in a modality-selective manner (Kim et al., 2016; Záborszky et al., 2018).

Sensory stimuli are processed differently against the background of diverse cortical states (Shimaoka et al., 2018; Poulet and Crochet, 2019). Ensembles of neighboring neurons depend on a certain level of desynchronization to encode complex features of stimuli by concerting the firing rate, spike timing, and the temporal order at which they fire (Hopfield, 1995; Montemurro et al., 2008; Kayser et al., 2009; Mohajerani et al., 2013; Luczak et al., 2015; Montijn et al., 2016). Increasing synchronization may lend structure to features, but can also obscure them (Fries et al., 2007; Pachitariu et al., 2015; Olcese et al., 2018). An intermediate level of arousal has been found to enhance the consistency and signal strength of encoded stimuli and is associated with optimal sensory processing (Polack et al., 2013; Schneider and Logan, 2014; Schölvinck et al., 2015; McGinley et al., 2015a; Olcese et al., 2018; Shimaoka et al., 2018). Cortical information processing is substantially shaped by the activity of diverse inhibitory interneurons and relies on a balanced interplay of excitatory and inhibitory inputs (Isaacson and Scanziani, 2011; Rubin et al., 2017). Cortical states can become altered regionally and globally upon “attention” to a stimulus (Olcese et al., 2018; Poulet and Crochet, 2019). This flexibility permits the mode of sensory processing to adapt to situational demands.

Such modes of attention, sensory processing, and integration have been described in terms of cortical oscillations of distinct wavelengths (delta, theta, alpha, beta, and gamma, as reviewed in Fries et al., 2007; Schroeder and Lakatos, 2009; Saalmann et al., 2012; Fries, 2015; McVea et al., 2016; Jensen et al., 2019; Sikkens et al., 2019). The underlying rhythmic synchronization of neural assemblies is largely produced by a synchronized spiking of inhibitory interneurons at higher frequencies (reviewed in Fries et al., 2007; Jensen et al., 2019). The high magnitudes of slower rhythms are generated by other mechanisms (see “Brain States Under Anesthesia” section; reviewed in Neske, 2015; Sanchez-Vives et al., 2017). Neurons that act in phase are more likely to fire together, since the active and refractory periods of presynaptic and postsynaptic neurons are aligned (Saalmann, 2014). This temporal coding scheme can facilitate or inhibit information transfer between local and distant cell groups by varying their phase relations.

Cortical oscillations can propagate as traveling waves of neural depolarization and spiking activity across the cortical surface (reviewed in McVea et al., 2016; Kuroki et al., 2018; Muller et al., 2018). Higher frequencies are thereby nested within slower waves, leading to rich patterns of background information onto which local events are processed. The distinct and complex spatiotemporal dynamics of these traveling waves (e.g., radial, planar, spiraling, or rotating) are coordinated by recurrent cortical networks and distant projections from thalamic and subthalamic nuclei (Bhattacharya et al., 2019). Anesthesia can affect neural information processing at both the local level (by setting an imbalance of inhibitory and excitatory inputs) and globally (by spoiling phase relations of cell ensembles and disrupting information transfer across brain areas).

### Brain States Under Anesthesia

Selecting an appropriate anesthetic protocol is in first place a question of choosing the right anesthetic class and dosage. Anesthetics currently used for murine fMRI (Table 1) can be divided into three classes based on their main molecular target receptors: γ-aminobutyric acid subtype A (GABA_A_) receptor agonists including volatile ethers, α2 adrenoreceptor (α2AR) agonists, and *N*-methyl-d-aspartate (NMDA) receptor antagonists. Each class exerts its sedating effects based on one of three key principles: (1) top-down by inhibiting cortical neurons directly (NMDA receptor antagonists); (2) bottom-up by suppressing subcortical arousal nuclei that affect cortical states (α2AR agonists); or (3) both, by inhibiting neurons across the entire brain (GABA_A_ agonists and volatile ethers; Figure 1).

**Table 1:** Anesthetic compounds used in mouse functional magnetic resonance imaging (fMRI).

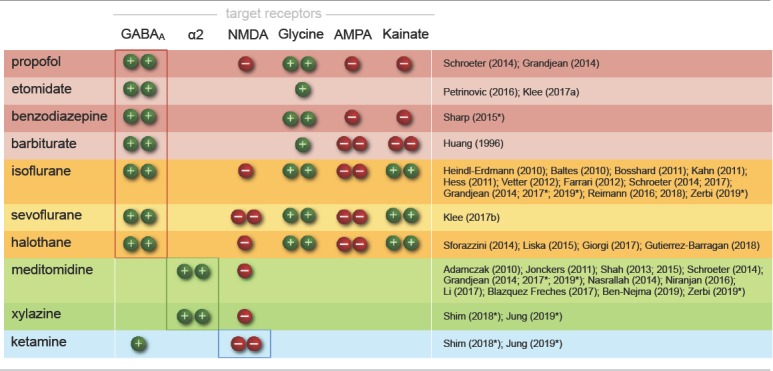

*Each drug addresses distinct receptor types by either agonistic (+) or antagonistic (−) modulation. Single or double symbols are used to indicate whether potentiation or inhibition is mild or strong. Anesthetics are grouped into three classes based on their main target receptors: GABA_A_ agonists, including injectable drugs and volatile ethers (red and yellow, respectively), α2AR agonists (green), and NMDA antagonists (blue). Alongside these key target receptors, the listed drugs modulate a host of further receptor types and may contribute to specific anesthetic effects (see, e.g., Alkire et al., 2008; Franks, 2008; McKinstry-Wu and Kelz, 2019). Halothane is chemically not an ether, yet it belongs to the group of halogenated vapors, like isoflurane and sevoflurane, which share largely overlapping target sites. Note that only drugs suitable for longitudinal studies are listed; the GABA_A_ agonists *α-chloralose* and *urethane* have also been applied in murine fMRI (Ahrens and Dubowitz, 2001; Xu et al., 2003, 2005; Grandjean et al., 2014; Schroeter et al., 2014) and will be discussed in “Sensory Processing and the Key Challenges in Murine fMRI” and “Functional Connectivity and Murine Resting-State fMRI” sections. References are shortened to first author (and year) to save space and allow for more comprehensive referencing. Asterisks indicate use in multimodal protocols*.

**Figure 2:**
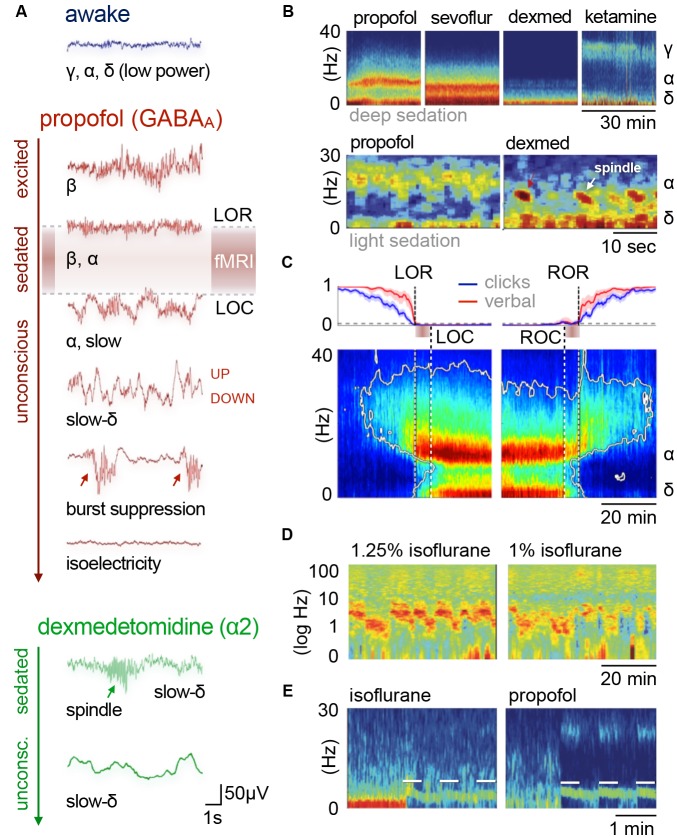
Cortical oscillatory signatures of brain states under anesthesia.** (A)** Changes in oscillatory signatures for increasing doses of propofol and dexmedetomidine measured from the human anterior cortex. With increasing anesthetic depth, oscillation frequencies decrease and synchronize. **(B)** Distinct oscillatory signatures induced by different anesthetics (upper panel) and dosages (lower panel). The spectrogram represents the spectrum of frequencies in a time and frequency domain, which facilitates the identification of structured frequency bands. **(C)** Loss of responsiveness (LOR) and connected consciousness (LOC) induced by increasing doses of propofol. LOR is defined by suppression of responses to click and verbal commands (upper panel). Note the occurrence of alpha bands and the absence of slow-delta bands in the phase between LOR and LOC in the spectrogram. The same is true for return of consciousness (ROC) and responsiveness (ROR). In rodents, alpha waves coincide with the loss of righting reflex (LORR), which corresponds to LOR, and slow-delta waves are indicated by a complete loss of movement (LOM), which corresponds to LOC. **(D)** Brain states may not be fully stable when maintained *via* anesthesia. Transitions between two or more intermediate states occurred over longer periods of constant isoflurane concentration in rats. This feature has been defined as metastability. Note the logarithmic scale used to highlight the transitions that occur primarily in the lower frequency band (0–10 Hz). Local field potentials (LFP) recording was conducted in the anterior cingulate cortex (ACC). **(E)** Stimulation (white bars) of dopaminergic neurons of the ventral tegmental area in the rat shifts cortical states under sedation from slow delta (<4 Hz) towards θ power (isoflurane), or towards θ and β power (propofol). *Oscillations*: slow (<1 Hz), δ (1–4 Hz), θ (4–8 Hz), α (8–15 Hz), β (15–30 Hz), awake-γ (30–80 Hz), ketamine-γ (25–35 Hz), spindle (9–16 Hz). Adapted with permission from **(A,B)** Purdon et al. (2015); (**B**, lower panel) Akeju and Brown (2017); **(C)** Purdon et al. (2013); **(D)** Hudson (2017), and **(E)** Solt et al. (2014).

Because their target sites differ, each anesthetic class produces distinct cortical oscillatory dynamics (Figures 2A,B; Ching and Brown, 2014; Purdon et al., 2015; Flores et al., 2017), which vary with the anesthetic dosage (Figures 2B,C). Most anesthetics within one class further exhibit variations in their cortical signatures as they act at multiple receptor types (Table 1), interfering with partially overlapping pathways. Scientific advances over the last decades have revealed an increasingly comprehensive, yet still incomplete picture of the underlying mechanisms (Steriade, 2000; Campagna et al., 2003; Alkire et al., 2008; Franks, 2008; Brown et al., 2010, 2018a; Akeju and Brown, 2017; Flores et al., 2017; Hemmings et al., 2019). Here, we briefly introduce current models on how these rhythms are generated by the effects of different anesthetics, and how the brain states that are indicated by distinct rhythms affect neural transmission and processing.

#### Thalamocortical Rhythmogenesis

**Figure 3:**
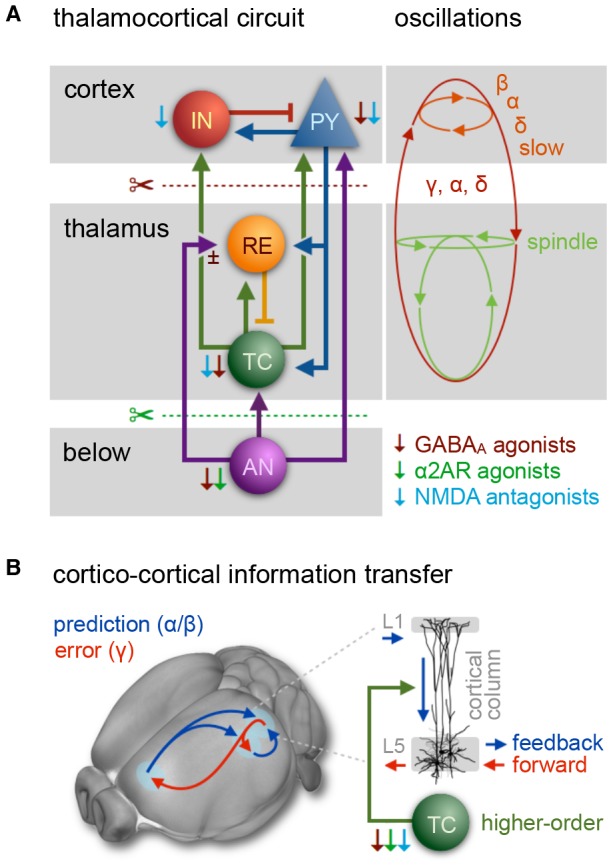
The thalamocortical circuit as wave generating unit, and cortical information transfer. **(A)** Scheme of thalamocortical circuit displays major interactions between GABAergic inhibitory interneurons (IN) and excitatory pyramidal cells (PY) in the cortex and thalamic GABAergic inhibitory reticular cells (RE) and excitatory thalamocortical neurons (TC). Arousal nuclei (AN) drive cortical desynchronization *via* cortical and thalamocortical circuits, including biphasic excitatory–inhibitory modulation (Sun et al., 2013). Anesthetic classes reduce the membrane potential of their respective target neurons (indicated by thin color-coded arrows), leading to inhibition and rebound spiking. Cortical oscillations are produced by synchronized periodic activity between two or more cell types. Transection below cortex (red scissors) leads to slow wave generation in the cortex; transection below thalamus (*cerveau isolé*, green scissors) leads to generation of sleep-like spindles and slow-delta waves. *Anesthesia-induced oscillations*: slow (<1 Hz), δ (1–4 Hz), α (8–15 Hz), β (15–30 Hz), γ (25–35 Hz), spindle (9–16 Hz). **(B)** Fronto-parietal information transfer along the cortical hierarchy. Feedback information (blue) is conveyed from higher-order frontal areas to primary sensory areas in the α/β band, feedforward information flow (red) from primary to higher-order areas in the γ band. In the predictive coding scheme feedback signals are considered predictive models of upcoming sensory states, whereas feedforward information represents the error resulting from a mismatch of a predictive model with an actual state. Feedback transmitted via long-range projections enters cortical columns at the dendritic trees of pyramidal cells in layer 1 (L1). The signal is transmitted to the soma of the same cells in layer 5, which requires driving input of higher-order TC nuclei. Feedback information transfer breaks down with the inhibition of higher-order TC nuclei at increasing depth of anesthesia. **(A)** Inspired by Ching and Brown (2014) and others. **(B)** Left panel: adapted from Sikkens et al. (2019). Note that visual higher-order areas in mice differ from those of primates, as reviewed in Glickfeld and Olsen (2017); Pennartz et al. (2019). Right panel: based on Suzuki and Larkum (2020).

The generation of cortical rhythms by anesthesia follows a common pattern for all anesthetics (as described in more detail below). All anesthetics exert their sedating effects by acting on specific target receptor types (Table 1), leading to inhibition and thus hyperpolarization of the affected neurons. Many types of excitatory and inhibitory neurons comprise specific membrane channels that open at distinct levels of hyperpolarization and lead to inward cation currents that cause the cells to quickly depolarize. Hence, the affected cells respond to strong inhibition with excitation, leading to *post-inhibitory rebound spiking*. These bursts of neural activity recur periodically, paced by the sum of inhibitory (hyperpolarizing) and excitatory (depolarizing) inputs that are required to exceed the threshold for rebound spiking. These inputs can be differently modulated by distinct anesthetic types and dosages. The resulting spiking patterns reverberate in recurrent neuronal circuits and can synchronize large networks of cortical neurons by reciprocal coupling, which manifests as cortical oscillations on EEG. These oscillations are generated either within the cortex or the thalamus, or between these two densely interconnected regions (Figure 3A; reviewed in McCormick and Bal, 1997; Steriade, 2001; Fuentealba and Steriade, 2005; Franks, 2008; Ching and Brown, 2014; Akeju and Brown, 2017).

Thalamic key players of rhythm generation are excitatory thalamocortical (TC) cells in intralaminar and medial thalamic nuclei (Baker et al., 2014; Flores et al., 2017). These higher-order TC nuclei receive and process input from cortical areas and serve as crucial relays of information transfer across the cortex (Theyel et al., 2010; Sherman, 2017; Mo and Sherman, 2019). Effective information transfer is thereby facilitated by frequency and phase synchronization of projection, relay, and target areas (Saalmann, 2014). In contrast, first-order TC nuclei primarily relay incoming sensory information to the cortex. First and higher-order TC nuclei undergo a major inhibitory impact of GABAergic thalamic reticular (RE) neurons, which project from the thin, outer shell of the thalamus—the thalamic reticular nucleus (as reviewed in Varela, 2014). RE neurons control TC traffic, synchronicity of firing patterns, and the level of arousal by targeted inhibition of TC cells (Sun et al., 2012). The level of depolarization in both TC and RE neurons is further regulated by afferents from cholinergic and monoaminergic arousal nuclei (Saper et al., 2010), which also modulate the membrane potential of cortical neurons: GABAergic inhibitory interneurons and glutamatergic excitatory pyramidal cells (Figure 3A).

#### GABA_A_ Agonists Suppress Neural Activity Across the Central Nervous System

Volatile ethers and GABA_A_ agonists such as isoflurane and propofol predominantly act on postsynaptic GABA_A_ receptors of excitatory neurons by increasing the preference of those ligand-gated chloride channels for the open state (Bai et al., 1999; Lee and Maguire, 2014). This enhances the inward chloride current and facilitates hyperpolarization and thus inhibition of the postsynaptic cell. Presynaptic actions that impair neurotransmitter release (Hemmings et al., 2005, 2019; Baumgart et al., 2015) further contribute to reducing neural activity across the central nervous system (CNS), including the cortex, thalamus, striatum, brainstem, and even the spinal cord (Figure 1B; Bowery et al., 1987; Hemmings et al., 2005; Brown et al., 2010; Phillips et al., 2018). This exerts hypnotic effects and gradually renders the animal unconscious in a dose-dependent manner (Akeju and Brown, 2017; Flores et al., 2017).

An initial excitement is often observed at the induction of anesthesia, accompanied by a disinhibition of motor activity and the emergence of relatively fast cortical oscillatory activity in the beta band (~13–30 Hz; Figure 2A; Ching and Brown, 2014; Le Van Quyen et al., 2016). The precise mechanisms responsible for this *paradoxical excitation* have yet to be fully explained. Network models indicate the involvement of hyperpolarization-triggered inward currents that cause cortical circuits of excitatory and inhibitory neurons to produce periodic firing in the beta range (McCarthy et al., 2008). With increasing inhibitory tone and sedation, beta rhythms slow down to alpha frequencies (Figures 2A, 3A; McCarthy et al., 2008; Baker et al., 2014; Flores et al., 2017). This is the onset of stable sedation marked by the loss of the righting reflex (LORR; Baker et al., 2014; Flores et al., 2017).

Although alpha oscillations (8–15 Hz) are produced within the cortex (Mukamel et al., 2014), the generation of temporally and spatially coherent alpha rhythms across larger areas requires the participation of the thalamus (Ching et al., 2010; Flores et al., 2017). The GABAergic inhibition of TC cells is thought to trigger enhanced post-inhibitory rebound spiking that is paced by the decay rate of inhibition, and thus depends on the anesthetic dosage (Figure 3A; Ching and Brown, 2014). Thalamic and cortical alpha activities reinforce each other by reciprocal coupling, resulting in strong TC synchronicity (Figure 3A; Ching et al., 2010; Ching and Brown, 2014; Crunelli et al., 2015; Bhattacharya et al., 2019).

Dense recurrent projections from the thalamus convey broad spatial coherence of alpha oscillations in the frontal cortex (Flores et al., 2017). In contrast, occipital thalamic projections of high-threshold TC neurons, which produce coherent alpha oscillations in the awake state are suppressed by a reduction of hyperpolarization-activated cation currents, presumably by secondary drug effects (Ching et al., 2010; Cimenser et al., 2011; Vijayan et al., 2013). This causes a characteristic spatial shift in EEG alpha power from the posterior to the anterior part of the brain, a phenomenon known as *anteriorization* (Akeju et al., 2014b).

LORR is likely caused by impaired information transfer and integration across cortical areas due to changing oscillatory modes. When sending and receiving cells are tuned in phase, postsynaptic neurons receive input during their active periods and are more likely to fire. Phase alignment facilitates neural transmission across distant cortical areas, which also involves higher-order TC nuclei (Saalmann, 2014; Sherman, 2017; Mo and Sherman, 2019). Higher-order TC nuclei are engaged with GABAergic drug-induced frontal alpha waves in humans and rodents (Ching et al., 2010; Liu et al., 2013a; Baker et al., 2014; Flores et al., 2017), which might affect phase–frequency relationships between sending and receiving areas and thus contribute to the disruption of cross-cortical communication.

However, the loss of awake-alpha (and higher frequency) waves has been shown to be a more reliable marker for stable sedation (Blain-Moraes et al., 2015; Pavone et al., 2017). A loss of awake-alpha is typical for the first stage of non-rapid eye movement (N1) sleep (Prerau et al., 2017) and was correlated with a lack of behavioral response to stimuli in N1 sleep (Prerau et al., 2014) and under light sedation *via* sevoflurane (Pavone et al., 2017).

Awake-alpha waves play an essential role in information transfer and integration across the cortex (Jensen et al., 2019; Senzai et al., 2019). For instance, neural activity in the alpha band in contralateral sensorimotor areas has been shown to phase lock robustly to somatosensory stimuli. If these stimuli are perceived consciously, the stimulus locking spreads rapidly to the frontal, parietal, and ipsilateral sensorimotor regions (Palva et al., 2005). For unperceived stimuli, the phase locking is weak and restricted to the initial sensorimotor area.

Synchronous alpha activity in frontal, parietal, and sensorimotor regions facilitates recurrent information transfer between these areas due to a phase alignment of neural activity in transmitting and receiving areas. Such alpha-phase synchrony between primary sensory and higher-order cortical areas is considered a neural basis of attention, and is strongly mediated by frontal and parietal regions. Fronto-parietal information transfer is involved in many higher-order functions, including the formation of higher-order functional networks in the awake, resting state (see “Functional Connectivity and Murine Resting-State fMRI” section; Coull, 1998; Kastner and Ungerleider, 2000; Rees et al., 2002; Zeman, 2004; Klimesch et al., 2007; Palva and Palva, 2007; Sadaghiani et al., 2012; Fries, 2015; Han et al., 2019; Sikkens et al., 2019).

Recurrent processing along the cortical hierarchy accounts for the contextual modulation of stimuli, including perceptual and semantic interpretation, endowing them with spatiotemporal context and behavioral significance (Pennartz et al., 2019). One function of recurrent processing is expressed in the *predictive coding framework*, in which the brain is constantly generating and updating sensory models of the world (as reviewed in Pennartz et al., 2019; Sikkens et al., 2019). Feedback projections from higher-order (e.g., prefrontal) to early sensory areas are thought to convey or facilitate predictive models of upcoming sensory states *via* alpha/beta oscillations. A mismatch of a predictive model with actual sensory input results in error signals that are presumably fed forward from early sensory to higher-order areas *via* gamma oscillations—through different, partially overlapping cortical layers—to update the model in a perpetual feedback/feedforward loop (Figure 3B, left panel; van Kerkoerle et al., 2014; Zhang et al., 2014; Bastos et al., 2015; D’Souza et al., 2016; Chao et al., 2018; Michalareas et al., 2016; Kissinger et al., 2018; Zhang et al., 2019).

Feedback and feedforward information transfer are considered cornerstones of effective signal processing in the conscious brain (as reviewed in Lamme, 2018; Pennartz et al., 2019). LORR can probably be attributed to impaired feedback or feedforward information transfer (Pavone et al., 2017; Sanders et al., 2018; Redinbaugh et al., 2020). Long-range feedback modulation, which in primary sensory areas promotes both alpha waves (Jensen et al., 2019; Sikkens et al., 2019) and desynchronized activity (Harris and Thiele, 2011; Ecker et al., 2016), undergoes a breakdown with increasing anesthetic depth (Imas et al., 2005b; Raz et al., 2014; Hentschke et al., 2017; Sanders et al., 2018; Murphy et al., 2019).

A recent cellular study revealed that cortico-cortical feedback flow through the cortical layers (mediated by L5 pyramidal cells) requires the driving input of higher-order TC nuclei (Figure 3B, right panel; Suzuki and Larkum, 2020). Since these higher-order TC nuclei in turn depend on the driving input of cholinergic projections from the brainstem (Figure 3A; Trageser et al., 2006; Masri et al., 2006, 2008), they appear to be particularly susceptible to the effects of various anesthetics. At already 1% isoflurane the inhibition of higher-order TC nuclei causes a disruption of cortico-cortical feedback loops.

This causal relationship unifies two central hypotheses of consciousness, which had previously assumed a dependence on either cortical feedback or higher-order TC loops (Alkire et al., 2008; Boly et al., 2011; Mashour, 2014). Also, feedforward signal propagation toward higher-order cortical regions has been reported to be increasingly suppressed with thalamocortical inhibition (Massimini et al., 2005; Sellers et al., 2015; Casarotto et al., 2016; Hentschke et al., 2017; Sanders et al., 2018; Redinbaugh et al., 2020). Therefore, studying sensory pathways within the cortex of anesthetized mice is often limited to brain areas that receive direct sensory thalamic or subcortical input.

Neural responses to sensory stimuli in primary cortices are largely preserved across sensory modalities (Lamme et al., 1998; Detsch et al., 1999; Pack et al., 2001; Imas et al., 2005a; Greenberg et al., 2008; Schumacher et al., 2011; Haider et al., 2013; Milenkovic et al., 2014; Raz et al., 2014; Sellers et al., 2015). Yet, even low anesthetic dosages alter the spatial and temporal structure of neural firing patterns, thereby disrupting information processing, which relies on precise timing of ensemble activity (Luczak et al., 2015; Yuste, 2015). For example, neural responses to visual stimuli in mice anesthetized with 0.25–1% isoflurane extend into larger V1 areas and are temporally prolonged, as they are less shaped by directed inhibition compared to the awake state (Haider et al., 2013; Sellers et al., 2015). This has also been observed for other drugs and sensory modalities (Devonshire et al., 2010).

#### Crossing the Borders: GABAergic Slow Waves and Burst Suppression

Increasing doses of anesthetics bring a complete loss of movement (LOM), which coincides with a sudden rise in slow-delta power; i.e., delta oscillations (1–4 Hz) primarily in the frontal areas, and slow waves (<1 Hz) across the entire cortex (Figure 2A; Steriade et al., 1993b, c, d; Flores et al., 2017; Chamadia et al., 2019). Slow-delta waves are characterized by alternations of persistent desynchronized network activity (depolarized “UP” states) and generalized neural silence (hyperpolarized “DOWN” states) of varying duration (Steriade et al., 1993b, c, d, 2001; Luczak et al., 2007). Slow-delta rhythms impose strong oscillatory dominance on the cortical firing patterns by nesting oscillations of higher frequencies in distinctive sequences (Steriade, 2001; Fuentealba and Steriade, 2005). Delta oscillations are grouped themselves by slow waves into larger sequences, although both waveforms share a very similar structure.

The exact phase-amplitude coupling of slower and faster waves is a function of the anesthetic depth (Chamadia et al., 2019). With light sedation, alpha waves lie in the trough of slow-delta oscillations. With increasing anesthetic depth, they experience a shift in phase until they ride on top of the slow waves’ peaks. This phase-amplitude syntax causes a functional decoupling of neural activity, and thus cortical communication to collapse in crucial networks, leading to deep anesthesia (see “Functional Connectivity and Murine Resting-State fMRI” section).

Cortical delta and slow waves persist following removal of the thalamus, although under such conditions, slow waves (at ~0.3 Hz) clearly dominate the EEG (Figure 3A, red scissors; Steriade et al., 1993c; Steriade, 2001). Slow waves even occur in small cortical pieces *in vitro* (e.g., in the primary visual cortex) and are robust to various experimental perturbations (as reviewed in Sanchez-Vives et al., 2017). Therefore, they have been proposed to represent the default activity pattern of cortical networks. The UP states bear striking resemblance to the desynchronized firing patterns of the awake state (Destexhe et al., 2007). The transition between the two micro-states originates from activity-dependent adaptation, which accumulates during UP states, attenuates neural transmission, and eventually switches to the DOWN state. In this state of neural silence (or strongly reduced activity), the network recovers its excitability until it elicits the next sudden transition to the UP state, forced up and amplified by the firing rates of neighboring neurons (Compte et al., 2003; Destexhe and Contreras, 2006; Braun and Mattia, 2010; Mattia and Sanchez-Vives, 2012; Neske, 2015; Sanchez-Vives et al., 2017).

Delta oscillations consist of cortical and thalamic components that are generated in overlapping higher-order TC nuclei along with alpha oscillations at increasing inhibitory tone (Steriade et al., 1993c, 1994; Amzica and Steriade, 1997; Steriade, 2001; Flores et al., 2017). Thalamic delta rhythmogenesis results from the interplay between two distinct hyperpolarization-activated currents within TC neurons, whereas GABAergic RE cells provide the hyperpolarizing input under natural conditions (Figure 3A; McCormick and Pape, 1990; Soltesz et al., 1991; Steriade et al., 1994). Since TC cells are not synaptically coupled, they fire in frequency, but not necessarily in phase (Amzica and Steriade, 1997; Neske, 2015). Coupling of the clock-like thalamic delta patterns is mediated through interconnected cortical pyramidal cells, which generate bursts at similar intrinsic delta frequencies, thereby synchronizing cortical areas *via* recurrent TC projections (Amzica and Steriade, 1997; Neske, 2015). While delta power rises coherently in anterior areas, slow waves occur all over the cortex but not necessarily in phase, leading to cortical fragmentation at moderate doses and thus impaired information transmission (Lewis et al., 2012).

An essential component in generating slow and delta oscillations is the inhibition of subcortical arousal nuclei, which causes cortical and TC neurons to lose their depolarizing driving input (Figure 3A; Steriade et al., 1991, 1993a). An inhibition of key arousal nuclei is indeed sufficient to explain delta and slow wave generation in sleep (reviewed in Weber and Dan, 2016) and quiet wakefulness (Neske, 2015). However, the pattern characteristics of slow waves under natural conditions are distinct from those elicited by GABAergic anesthesia, which are produced under additional direct thalamic and cortical inhibition (Chauvette et al., 2011; Kenny et al., 2014; Busche et al., 2015; Akeju and Brown, 2017; Arena et al., 2017; Aggarwal et al., 2019). The full inhibitory package results in enhanced suppression of spontaneous desynchronized cortical background activity, which causes successively quiescent networks in the DOWN state with increasing anesthetic depth.

**Figure 4:**
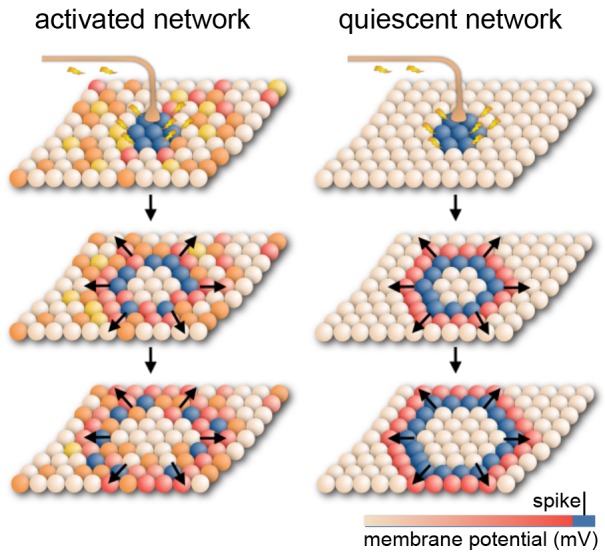
Model for cortical signal propagation by nearest neighbor recruitment. In an activated cortical network with desynchronized background activity (as observed in normal waking states), local stimulation elicits waves that weakly entrain neuronal spiking as they travel across the network. In a quiescent cortical network with almost no background activity (as in deeply anesthetized states) local stimulation elicits dense traveling waves, which recruit nearly all cells as they pass. Spheres represent neurons whose membrane potential is indicated by color. Based on Muller et al. (2018).

Such quiescent cortical networks of synchronous activity have been proposed to be ideal substrates for the propagation of dense traveling waves (Figure 4; Muller et al., 2018). Given that all neurons act in phase, neurotransmission reaches all post-synaptic cells during episodes of increased excitability. Triggering UP states in this low complexity environment instantly recruits neighboring neurons *via* massive recurrent excitation (Sanchez-Vives et al., 2017), so that even local events (like sensory inputs) can elicit dense traveling waves that entrain nearly all cells as they pass (Muller et al., 2018).

Such dense, traveling slow waves have been found to spread across the cortical surface, irrespective of anatomical boundaries, like a set of waves created by a drop in an oil bath (Figure 4; Massimini et al., 2004, 2007; Luczak et al., 2007; Stroh et al., 2013; Sanchez-Vives et al., 2017; Muller et al., 2018). Whether these waves remain local or propagate depends on the (local) brain state or desynchronization (Massimini et al., 2004, 2007; Nir et al., 2011; Vyazovskiy et al., 2011; Schwalm et al., 2017; Muller et al., 2018) and hence on anesthetic depth.

Aside from functional deafferentation and cortical inhibition, which provides a powerful substrate for long-range spread by nearest-neighbor recruitment, the synchronizing drive from the thalamus may add to the manifestation of slow waves (Crunelli and Hughes, 2010; Sheroziya and Timofeev, 2014; Crunelli et al., 2015; Neske, 2015). For the sake of translatability, functional brain studies should be generally performed at lower dosages than those leading to the dominance of slow wave and delta power, which affects neuronal excitability and stimulus–response properties (see “Anesthetic Depth and Consciousness—the Virtue of Translational Neuroimaging” section; Steriade et al., 1993b, c; Petersen et al., 2003; McGinley et al., 2015b).

Higher anesthetic dosages lead to *burst suppression*-characterized by increasingly prolonged “DOWN” micro-states alternating with periods of brief bursts of spikes and waves (Figure 2A; Steriade et al., 1994; Lewis et al., 2013; Amzica, 2015; Purdon et al., 2015). This is an increasingly hyperexcitable state in which large networks of cortical neurons suddenly discharge in tight synchrony (Steriade et al., 1994; Kroeger and Amzica, 2007; Ferron et al., 2009). Subsequently they fall into a post-burst refractory period, due to metabolic exhaustion (Hirsch and Taylor, 2010; Ching et al., 2012; Ching and Brown, 2014). This period of complete cortical silence is induced by adenosine triphosphate (ATP)-sensitive cation channels, which open when ATP decreases below critical levels (Cunningham et al., 2006). The resulting cation influx renders neurons unable to produce action potentials. Upon metabolic regeneration, the channels close again, and cortical networks gradually regain hyperexcitability by depolarizing, until they exhibit the next discharge (Kroeger and Amzica, 2007). Hyperexcitability is generated within the cortex as a result of the functional impairment of cortical afferents (Echlin et al., 1952; Henry and Scoville, 1952; Hughes, 1986; Niedermeyer et al., 1999; Kenny et al., 2014; Amzica, 2015).

Thalamic sensory transmission is preserved during isoelectric and burst episodes, although cortical discharge rates are substantially reduced during isoelectric periods (Detsch et al., 2002). During periods of cortical hyperexcitability, even subliminal stimuli are sufficient to trigger bursting activity (Yli-Hankala et al., 1993; Hartikainen et al., 1995; Hudetz and Imas, 2007; Kroeger and Amzica, 2007; Amzica, 2009). Spontaneous and evoked bursts can spread across large cortical areas (Steriade et al., 1993b; Timofeev et al., 2000; Land et al., 2012) and may further engage remote hippocampal (Sirota et al., 2003; Hahn et al., 2006; Ji and Wilson, 2007; Busche et al., 2015) and thalamic structures (Steriade et al., 1993c; Stroh et al., 2013; Sheroziya and Timofeev, 2015), presumably by excitatory projections (Leong et al., 2016). In this regard, burst suppression events can be considered as non- or quasi-periodic slow waves with a long refractory time and high discharge.

Further increasing the anesthetic dosage leads to longer suppression, shorter burst periods, and less reactivity to stimuli (Hartikainen et al., 1995), which culminates in complete neural inexcitability and finally isoelectricity due to increasing (thalamo)cortical and subcortical suppression (Figure 2A; Kroeger and Amzica, 2007).

#### α2AR Agonists Induce a Sleep-Like State by Suppressing General Arousal

Sympatholytics or α2AR agonists such as xylazine, medetomidine, or its potent dextro enantiomer dexmedetomidine exert their sedating and antinociceptive effects primarily by acting on presynaptic α2_A_ adrenergic receptors of noradrenergic cells that project from the locus coeruleus. This results in a hyperpolarization of the affected neurons and a reduction in the release of noradrenaline to their target sites (Correa-Sales et al., 1992; Jorm and Stamford, 1993; Chiu et al., 1995; Van Bockstaele et al., 1999). The locus coeruleus is a major arousal nucleus that projects to the basal forebrain (a subcortical arousal structure, which regulates cortical states by cholinergic efferents; Nelson et al., 2005; Hoover and Vertes, 2007; Pal et al., 2018), intralaminar nucleus of the thalamus, thalamic reticular nucleus, preoptic area of the hypothalamus, and diffusely into the cortex (Figure 1C; Asanuma, 1992; Nelson et al., 2003; Samuels and Szabadi, 2008a; Saper et al., 2010; Zhang et al., 2015; Fu et al., 2017; Brown et al., 2018a). Nociceptive pathways are further affected by the direct activation of α2 receptors in the spinal cord (Andrieu et al., 2009).

Decreased noradrenaline release in the preoptic area of the hypothalamus causes disinhibition (and thus excitation) of local endogenous sleep-promoting cells that send inhibitory projections to other key arousal nuclei in the midbrain and pons (Figure 1C; Sherin et al., 1998; Saper et al., 2005; reviewed in Saper et al., 2010; Scammell et al., 2017). This causes an inhibition of widely-projecting neurons in these arousal nuclei, which in turn decreases the depolarizing input to thalamic and cortical areas, leading to sleep-like, spindle (9–16 Hz), and slow-delta (0.1–4 Hz) oscillations. This pattern is distinct from that induced by GABA_A_ agonists due to the lack of direct cortical and thalamic inhibition (Figures 2A–C, 3A; Noreika et al., 2011; Baker et al., 2014; Nasrallah et al., 2014a; Akeju and Brown, 2017; Banks et al., 2017).

Similar sleep-like states were observed following a transection below the thalamus in cats, the so-called *cerveau isolé* (the isolated cerebrum; Figure 3A, green scissors; Bremer, 1935; Steriade et al., 1993c). This dramatically demonstrates that the disconnection of thalamic and cortical circuits from the input of subcortical arousal nuclei suffices to produce oscillatory and spiking patterns, similar to non-REM sleep at the N2 stage and sedation elicited by α2AR agonists (Akeju and Brown, 2017).

Under light sedation, spindle rhythms occur in brief bursts of ~0.5–3 s (Figures 2A,C; Baker et al., 2014; Nasrallah et al., 2014a; Purdon et al., 2015). Spindles are generated within the thalamic RE nucleus, which is considered the *spindle pacemaker* (Steriade et al., 1985; Halassa et al., 2011; Kim et al., 2012). Evidence for this includes the deafferentation of this structure from the cortex and the remaining thalamus, upon which the nucleus continues to generate spindles (Steriade et al., 1987). Rhythmogenesis thereby strongly depends on the level of hyperpolarization of thalamic RE neurons and the intactness of their long and thin dendrites, which are richly endowed with low-threshold hyperpolarization-triggered Ca^2+^ channels (as reviewed in Fuentealba and Steriade, 2005; Crandall et al., 2010; Astori et al., 2011; Zaman et al., 2011).

Suppression of arousal nuclei leads to decreased depolarizing input to RE cells (McCormick, 1992; Saper et al., 2010; Sun et al., 2013), which can cause hyperpolarization below the threshold, leading to strong dendritic Ca^2+^ spikes. This results in depolarization and rhythmic bursts of action potentials that are sustained by additional voltage-gated cation channels (reviewed in Fuentealba and Steriade, 2005; Lüthi, 2014). GABAergic transmission and electrical coupling in recurrent networks of RE neurons are sufficient to synchronize oscillations in the range of spindles (Bazhenov et al., 1999; Fuentealba and Steriade, 2005).

In the intact brain, the initiation and synchronization of spindles is supported by TC and cortical circuits. Glutamatergic stimuli can easily trigger low-threshold Ca^2+^ spikes in RE cells (Crandall et al., 2010). Therefore, spindles can be initiated by diverse inputs, including spontaneous oscillating TC cells or cortical volleys that impinge on RE networks (Figure 3A; Destexhe et al., 1996). For example, the cortical transitions to the UP state of slow waves may be quickly followed by a resulting spindle wave, which is a common sequence in slow wave sleep, known as the *K-complex* (Steriade et al., 1993b; Amzica and Steriade, 1997). Notably, K-complexes have not been described for sedation through α2AR agonists (Huupponen et al., 2008; Nasrallah et al., 2014a; Akeju et al., 2016a).

A burst of action potentials in an RE neuron causes hyperpolarization, rebound spiking and thus neural firing in multiple TC neurons; this reverberates in large thalamic networks by recurrent synapsing (Figure 3A; Bazhenov et al., 1999; Beenhakker and Huguenard, 2009). The strong synchronized rhythms of TC neurons can further entrain cortical pyramidal cells and interneurons in both prefrontal and sensory cortices (Peyrache et al., 2011). Cortico-cortical recruitment may cause further synchronization (Kandel and Buzsáki, 1997). The short spindle episodes are terminated *via* intrinsic ionic mechanisms in both thalamic RE and TC cells that are triggered by high concentrations of accumulated intracellular Ca^2+^ (alongside other strategies reviewed in Lüthi, 2014).

Delta waves resemble spindles in that they do not appear continuously in sleep or for light α2AR-induced sedation (Figure 2B; Steriade et al., 1993a, c; Baker et al., 2014). This changes at higher anesthetic dosages through a further suppression of depolarizing arousal inputs to thalamic RE neurons; they further hyperpolarize, and their firing patterns subsequently change from spindle to delta waveforms (Figures 2A,B; Nuñez et al., 1992; Destexhe et al., 1994).

The transition to delta rhythms is mirrored in TC circuits and entrains cortical networks. The result is continuous delta oscillation in thalamic and cortical areas, whose onset has been reported to coincide with a discrete drop in frequency at the instant of dexmedetomidine-induced LORR (Baker et al., 2014). This is accompanied by a significant phase shift of delta waves in the central medial thalamus as compared to cortical areas. Such phase shifts in higher-order TC nuclei can disrupt cortico-thalamo-cortical communication, causing a breakdown of information transfer between cortical areas (Slézia et al., 2011; Saalmann, 2014; Mo and Sherman, 2019; Suzuki and Larkum, 2020) and thus LORR.

First-order thalamic relay nuclei may not participate in producing continuous delta oscillations (Baker et al., 2014). Instead, they intensify spindle generation following LORR. The transient inhibition of TC neurons during spindle periods has been reported to prevent them from transferring sensory information to the cortex (Steriade and Contreras, 1995; Fuentealba and Steriade, 2005). Accordingly, spindle density correlates with the gating of sensory inputs in sleep (Dang-Vu et al., 2010; Wimmer et al., 2012; Chen et al., 2016).

However, in α2AR-induced sedation, primary sensory routes remain largely intact. Medetomidine has been shown to preserve cortical responsiveness to acoustic stimuli in primary sensory areas in rats (Banks et al., 2017). Subcutaneous electrostimulation of the paw has been reported to evoke slightly reduced potentials in the primary somatosensory cortex (S1) compared to GABAergic drugs (Hayton et al., 1999; Li et al., 2003). Yet, the amplitudes of somatosensory evoked potentials in S1 are not affected with increasing concentrations of medetomidine (Li et al., 2003; Nasrallah et al., 2014a). Spindle activity in the paw region of S1 occurs at medetomidine concentrations that are commonly used for fMRI (Nasrallah et al., 2014a).

The induction of LORR at continuous delta activity has been demonstrated in a study that kept rats in a rotating tube in which they had to constantly adapt their position until they rolled onto their sides and remained supine (Baker et al., 2014). Such constant active behavior increases the activity of arousal nuclei (Marlinski et al., 2012; Furth et al., 2017) and interferes with the “sleep-like” sedative state elicited by α2AR agonists (Kamibayashi and Maze, 2000; Venn and Grounds, 2001). In fact, sedation induced by α2AR agonists is far more arousable than for GABAergic drugs (see “Sensory Processing and the Key Challenges in Murine fMRI” and “Functional Connectivity and Murine Resting-State fMRI” sections; Noreika et al., 2011; Sanders et al., 2012; Akeju and Brown, 2017; Banks et al., 2017). Thus, higher anesthetic dosages were required to induce LORR in actively moving rats (Baker et al., 2014) than to induce the sedation sufficient for an fMRI experiment (Nasrallah et al., 2014a). For the GABAergic drug propofol, LORR occurred with the onset of alpha waves both in rotating tubes (Baker et al., 2014) and in the freely resting rodent (Flores et al., 2017).

#### NMDA Receptor Antagonists Primarily Affect Cortical Neural Activity

Ketamine is an NMDA receptor antagonist that provides dissociating, quasi-hypnotic effects by selectively binding and blocking NMDA receptors that are primarily expressed in cortical inhibitory and excitatory neurons, but also in thalamic TC and RE cells (Deleuze and Huguenard, 2016), and to a lesser extent in subcortical arousal nuclei and peripheral nerves (Gunduz-Bruce, 2009). Ketamine generates opposing effects in a dose-dependent manner. At lower concentrations, it binds preferentially to NMDA receptors on cortical GABAergic inhibitory interneurons, which show about 10-fold higher sensitivity to NMDA blockade than pyramidal neurons (Grunze et al., 1996).

NMDA receptor blockage prevents cation influx and depolarization, resulting in decreased GABAergic transmission to downstream excitatory neurons (Homayoun and Moghaddam, 2007; Seamans, 2008). Thus, excitatory neurons are disinhibited and become depolarized (Figure 1D, Brown et al., 2011; Phillips et al., 2018; Picard et al., 2019). An appropriate depolarization of cortical pyramidal neurons can elicit fast oscillations in the lower gamma band (Steriade et al., 1991; Nuñez et al., 1992; Gray and McCormick, 1996), synchronized by rhythmic inhibition (Buzsáki and Chrobak, 1995; Olufsen et al., 2003; Börgers et al., 2005).

The fast rhythm generation is promoted by ketamine actions in thalamic circuits, which switches the firing patterns of TC and RE neurons from the burst mode to the tonic generation of single action potentials (Anderson et al., 2017; Mahdavi et al., 2019). Such synchronous fast oscillations in the gamma band (25–80 Hz) typically emerge with cortical processing during higher-level mental activity, and in REM sleep, which is associated with dreaming mentation (Llinás and Ribary, 1993).

Low-dose ketamine elicits gamma waves in a narrow frequency band of ~25–35 Hz and diffuse excitatory cortical activity (Akeju et al., 2016b). At this stage, hallucinations, dissociated states, euphoria, and dysphoria have been reported in clinical use: they have been attributed to preserved communication across brain areas at low inhibitory modulation and control, as well as a disruption of dopaminergic neurotransmission in the prefrontal cortex (Moghaddam et al., 1997; Purdon et al., 2015).

At higher doses, ketamine increasingly begins to block NMDA receptors at excitatory pyramidal neurons, causing cortical inhibition to predominate. It further suppresses arousal pathways by blocking excitatory projections from the parabrachial nucleus and from the medial pontine reticular formation in the brainstem to the thalamus and to the basal forebrain (Boon and Milsom, 2008; Fuller et al., 2011; Brown et al., 2018a). In turn, gamma waves become interspersed with slow-delta oscillations (Figure 2B; Ruiz-Mejias et al., 2011; Akeju et al., 2016b), which are augmented by direct drug action in the thalamus (Kiss et al., 2011; Zhang et al., 2012). The suppression of higher-order TC loops (Suzuki and Larkum, 2020) and a breakdown of cortical coherence is considered likely to be the mechanism that induces LORR (Pal et al., 2015; de la Salle et al., 2016; Schroeder et al., 2016; Brown et al., 2018a).

Ketamine can cause regional hypo- and hyperactivation across the cortex (Porro et al., 2004) and introduces further complexity to the processing of external stimuli (Oye et al., 1992; Zandieh et al., 2003; Schwertner et al., 2018). Besides antinociception mediated by direct inhibition of peripheral nociceptive afferents expressing NMDA receptors (Sinner and Graf, 2008), ketamine has also been shown to persistently reduce aversive responses to noxious stimuli in a top-down manner by prolonged suppression of hyperactive neurons in the anterior cingulate cortex (ACC) in rodent chronic pain models (Zhou et al., 2018). Low dosages increase SEP in rats and mice, even compared to the awake state (Franceschini et al., 2010; Michelson and Kozai, 2018) although the degree depends on the mouse strain (Maxwell et al., 2006). SEP from the cortex to higher-order TC neurons are decreased, illustrating a disturbed functional state of cortico-thalamo-cortical and thus cortico-cortical circuits (Anderson et al., 2017). Low-dose ketamine increases power in the gamma band, but also delta power can increase more significantly than for low-dose isoflurane (Michelson and Kozai, 2018).

### Metastability of Brain States, Hysteresis, and Behavioral Monitoring

The comparison of brain states induced by various anesthetic compounds and dosages illustrates the diversity of mechanisms that lead to sedation. Whether an adjusted brain state can be stably maintained throughout the entire duration of an fMRI session remains to be clarified. Volatile ethers and intravenously injectable anesthetics appear to permit relatively stable maintenance of cortical oscillation patterns (Figures 2B,C; Purdon et al., 2013, 2015; Flores et al., 2017). However, transitions between two or more brain states have been observed over longer periods (1 h) at fixed concentrations of isoflurane in rats (Hudson et al., 2014). The transitions occurred predominantly in the lower-frequency band and could be well observed when the data were expressed on a logarithmic scale (Figure 2D).

The authors referred to this condition as “metastable” or potentially “multistable,” given that intermediate brain states shifted between two or more *attractors* distributed in phase space (for further discussion, see Breakspear, 2017; Hudson, 2017). There is evidence that such meta- or multistability is a general feature of brain states under anesthesia for various compounds and across species, although studies dedicated to detail such transitions over long periods are sparse (reviewed in Hudson, 2017). To what extent the transition of brain states may jeopardize the concordance of results across experiments (e.g., by exerting significantly different effects on signal processing) remains an open question, and has to be established for specific anesthetic protocols applied.

The reported state transitions were observed during recovery of consciousness; after an initial concentration of 1.75% isoflurane for 1 h, which reliably produced burst suppression, the concentration was reduced by 0.5%, maintained at that level for 1 h, and the process was repeated over a total of 6 h. Given this design, the dynamics of metastability were likely swayed by neural inertia—an intrinsic feature of neural circuits to resist swift transitions between consciousness and unconsciousness (Friedman et al., 2010; Proekt and Hudson, 2018; Proekt and Kelz, 2018). During recovery of consciousness neural inertia tends to trap the brain in an unconscious state. Due to this “stickiness,” a lower anesthetic dosage is required to maintain a similar anesthetic depth for the emergence from unconsciousness, compared to the induction of anesthesia (Hudson et al., 2014; Hudson, 2017). This dependence of the brain state on its history (hysteresis effect) cannot be explained by pharmacokinetic actions (see “Functional Connectivity and Murine Resting-State fMRI” section; Kelz et al., 2008; Friedman et al., 2010), and should be considered in preclinical studies for which an initial bolus induction of anesthesia is common practice.

Another issue in maintaining an intermediate brain state throughout an fMRI experiment might arise from nociceptive or stressful stimuli. Clinical experience and preclinical studies have shown that noxious stimuli, as well as direct stimulation of key arousal nuclei, can shift cortical states from slow synchronized towards highly desynchronized oscillations (Figure 2E; Hudetz et al., 2003; Solt et al., 2014; Vazey and Aston-Jones, 2014; Akeju and Brown, 2017; Sanders, 2017; Pal et al., 2018; Hayat et al., 2019). When the activity of the arousal-related nuclei exceeds a certain level, the animal wakes up from light anesthesia, and falls back into sedation when the activity subsides (Figure 2E; Solt et al., 2014). Consequently, an anesthetic protocol should be tailored, in terms of class and dosage, to the experimental task at hand. If the aim of a study is to assess functional connectivity (FC) at rest, there is no need to adjust the anesthetic depth so that the mouse remains unresponsive to nociceptive stimuli.

This calls into question the idea of the minimum alveolar concentration (MAC) in rodent fMRI—a well-established behavioral measure of anesthetic depth for volatile ethers (Steffey, 2017). Briefly, a MAC of 1.0 is defined as the average of the lowest anesthetic concentration that prevents a behavioral response upon a standardized pain stimulus, and the highest concentration that still permits a nocifensive response in 50% of tested subjects (Eger et al., 1965; Quasha et al., 1980). A MAC of 0.7 in rats has been reported to suppress the righting reflex and active attempts to withdraw; at a MAC of 0.3 (“MAC-awake”), frequent movements of the snout including sniffing, chewing, licking, and gross limb movements still occurred (Hudetz, 2002). However, these behavioral markers are not dependent on the application of any nociceptive stimulus that is not applied in the actual experiment.

Anesthetic depth can be monitored by behavioral markers and pupil dilation diameter to assess the level of arousal (Erisken et al., 2014; McGinley et al., 2015a; Joshi et al., 2016; Reimer et al., 2016; Binda and Gamlin, 2017; Shimaoka et al., 2018). Behavioral observation is indispensable to determine the level of sedation and immobilization of an animal, although the capacity to infer the actual brain state from these responses is limited (Pal et al., 2018). Hence, it is highly encouraged to adjust and compare anesthetic protocols based on oscillatory signatures and neural response properties outside the MR environment. Behavioral monitoring becomes increasingly crucial as different anesthetic classes and combinations of drugs are employed to sedate the animal (see “Multimodal Anesthesia in Translational fMRI” section).

### Anesthetic Depth and Consciousness—the Virtue of Translational Neuroimaging

In general, anesthetic mechanisms are highly conserved across species (Achermann and Borbély, 1997; Steriade et al., 2001; Mölle et al., 2002; Eschenko et al., 2006; Destexhe et al., 2007; Buzsáki and Moser, 2013; Shein-Idelson et al., 2016) and the oscillatory signatures of humans and mice are virtually similar upon applications of specific anesthesia and stages of anesthetic depth (Seth et al., 2005; Flores et al., 2017; Guidera et al., 2017; Hudson, 2017; Storm et al., 2017; Olcese et al., 2018). This relation provides a rough means with which to interpret brain states in terms of perceived anesthetic depth, stress, or pain. In humans, specific brain states can be linked to the introspective experience, as revealed by subjective reports. Using oscillatory signatures, a third-person observer can precisely specify the moment at which the “lights switch off,” from the first-person perspective (Purdon et al., 2015; Brown et al., 2018b). For GABAergic drugs, this is the emergence of slow-delta oscillations (Figure 2C; Purdon et al., 2013, 2015).

Traditionally, this transition point is considered to be the “loss of connected consciousness” (LOC; Sanders et al., 2012) and is defined as a loss of sensory perception and interoception—the perception of internal processes, including pain, anxiety, stress, discomfort, or sense of time (Purdon et al., 2013, 2015; Warnaby et al., 2016; Sleigh et al., 2018; Chamadia et al., 2019). This is distinguished from “disconnected” phenomenal consciousness, like the awareness of pure darkness (Sanders et al., 2012; Sleigh et al., 2018) or dreaming, although dreams have often been reported for medetomidine-induced LOC (Akeju and Brown, 2017; Mashour and Hudetz, 2017, 2018).

In the state of propofol-induced LOC, innocuous sensory stimuli such as words or tones could no longer be perceived, and no hemodynamic responses to these stimuli were detected using fMRI (Ní Mhuircheartaigh et al., 2013; Warnaby et al., 2016; Lichtner et al., 2018). However, BOLD patterns in response to nociceptive stimuli were partially preserved (Lichtner et al., 2018). These findings imply that the oscillatory signatures associated with LOC may be useful as a marker delineating the lower limit of anesthetic depth in sensory fMRI (Figures 2A,C).

Another transition point can be determined based on behavioral observation—the “loss of behavioral responsiveness” (LOR), which occurs earlier, approximately with the appearance of frontal alpha oscillations and before slow waves dominate the EEG (Figure 2C; Purdon et al., 2013; Warnaby et al., 2016). At this point, subjects fail to exhibit volitional responses to sensory or even noxious stimuli (Sanders et al., 2012; Ní Mhuircheartaigh et al., 2013; Purdon et al., 2013; Warnaby et al., 2016; Sleigh et al., 2018). Stimuli are still perceived and processed when they are applied, as indicated by fMRI data and subjective reports (Warnaby et al., 2016). Volunteers described this as a state of detachment from the stimuli and from “themselves”—i.e., they were not fully unconscious, but also not aware that the stimuli were related to them. Tones that were presented rather subtly and very briefly (1 kHz, 60 ms) were not sufficient to elicit significant BOLD responses beyond the thalamic relay nuclei. The BOLD patterns evoked by words and nociception, on the other hand, were reported to reflect closely those of the conscious state, with substantially lower activity in only the right dorsal anterior insular cortex (Warnaby et al., 2016). This area is reportedly associated with body ownership and self-agency (Warnaby et al., 2016; Lichtner et al., 2018; Sleigh et al., 2018). The anesthetic inhibition of the anterior insula and related interoceptive networks led the authors to reformulate their understanding of the hypnotic effects of anesthesia as a gradual disruption of “selfhood,” which occurs with an increase of anesthetic depth, finally leading to oblivion (Sleigh et al., 2018).

This offers not only a direct link between introspective reports, behavioral and neuroimaging markers, but also a vivid example of how the examination of large-scale networks using fMRI can complement models of LOC that are based on the suppression of feedback or feedforward information transfer along the cortical hierarchy (see “GABA_A_ Agonists Suppress Neural Activity Across the Central Nervous System” section; reviewed in Pennartz et al., 2019; Sikkens et al., 2019). Because similar transitions in oscillatory signatures can be observed in humans and rodents, maintaining and stabilizing a brain state between LOR (corresponding to LORR in animals; Baker et al., 2014; Banks et al., 2017; Flores et al., 2017) and LOC (corresponding to LOM in animals, and the occurrence of slow-delta waves; Flores et al., 2017) could be an attractive target state in which to perform fMRI in mice, at least for sensory perception tasks (Figures 2A,C). Hemodynamic coupling does not appear to be directly affected by propofol (Veselis et al., 2005). However, potential vasomodulatory effects of anesthesia have to be taken into account when inferring dose-dependent suppression of neural activity based on fMRI (Table 2; Aksenov et al., 2015).

**Table 2:** Effects on hemodynamic integrity of anesthetics used in mouse fMRI.

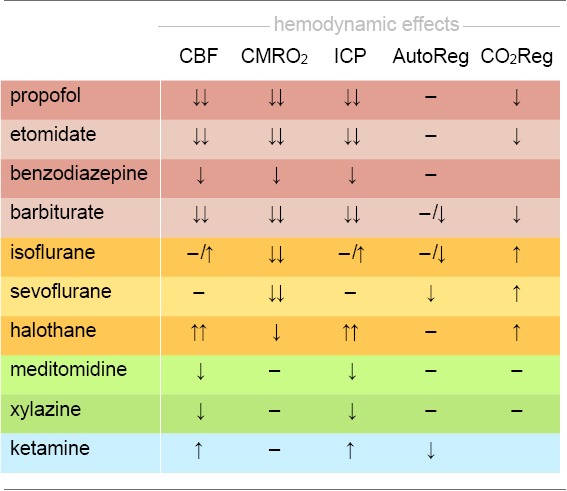

*Anesthetics are listed according to their classes: GABA_A_ agonists, including injectable drugs (red) and volatile ethers (yellow), α2AR agonists (green), and NMDA antagonists (blue). Vascular effects including vasodilation and vasoconstriction may impact on cerebral blood flow (CBF), intracranial pressure (ICP), or cerebral autoregulation (AutoReg). Drugs may further affect the cerebral metabolic rate of oxygen consumption (CMRO_2_) and the regulation of the CO_2_ metabolism (CO_2_Reg). Effect: ↓ reduction; ↓↓ strong reduction; ↑ increase; ↑↑ strong increase;–no change. Based on Bittner et al. (2019)*.

## Murine fMRI and Hemodynamic Integrity

Research on murine fMRI has grown exponentially from only a handful of publications in the first decade (Huang et al., 1996; Ahrens and Dubowitz, 2001; Mueggler et al., 2003; Xu et al., 2003, 2005) to more than 50 in the following. Nevertheless, anesthesia in murine fMRI presents a multitude of challenges beyond the determination of functional brain states and neural response properties, and anesthetic protocols are still far from being effectively tailored to meet murine physiological and hemodynamic demands. Preserving hemodynamic integrity that approximates a physiological state is essential to exploit the two key advantages of murine fMRI: non-invasively investigating whole-brain functional dynamics, and the opportunity to directly compare these dynamics across species, including humans. The experiments that have been performed can be classified into at least four categories: (1) sensory perception; (2) nociception and pain; (3) FC based on the resting state; and (4) within-brain stimulation involving the use of opto- and chemogenetics. Each category reveals particular aspects of anesthetic effects in fMRI and deserves consideration in terms of the requirements for anesthetic protocols that are appropriate to obtaining neuroimaging data of translational value.

### Sensory Processing and the Key Challenges in Murine fMRI

Sensory studies were among the earliest and most elementary applications of murine fMRI. Nevertheless, only a handful of publications report on innocuous sensory stimulation tasks that address the “natural” sensory perception routes of primary organs—like eyes, ears, nose, skin, or whiskers—using various anesthetic protocols. The very first mouse fMRI study (Huang et al., 1996) concerned visual perception and applied the GABA_A_-positive allosteric modulator and agonist pentobarbital; subsequent olfactory studies used urethane (Xu et al., 2003, 2005). Later visual (Niranjan et al., 2016) and auditory (Blazquez Freches et al., 2018) studies applied the α2AR agonist medetomidine, and deflection of the vibrissae was conducted under low-dose (0.5–1%) isoflurane (Kahn et al., 2011). All these studies identified modality-relevant sensory pathways, including primary sensory and partially preserved secondary cortical and thalamic structures. Higher-order or association areas have not been reported.

#### Technical Detection Limit

**Figure 5:**
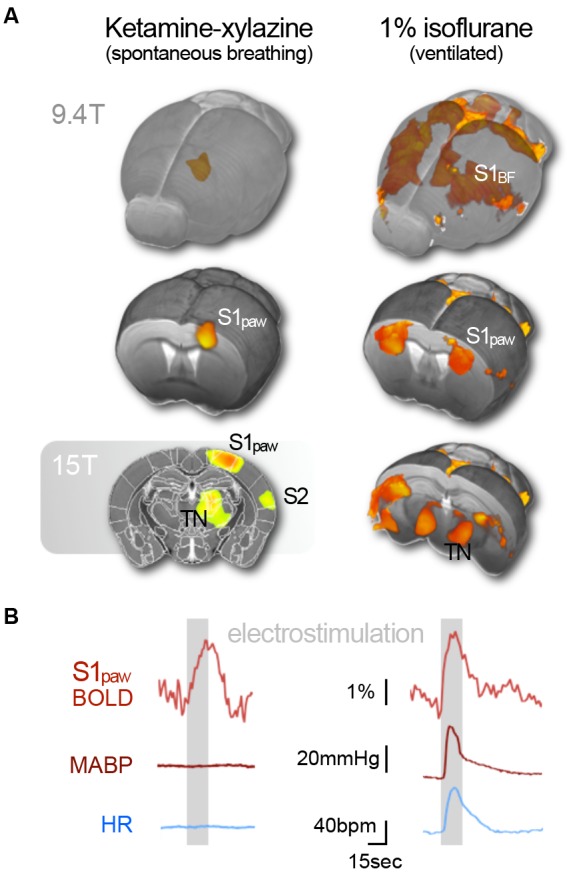
Subcutaneous electrostimulation of the paw in spontaneous breathing and mechanically ventilated mice under two different anesthetic regimens. **(A)** Blood oxygenation level-dependent (BOLD) patterns show unilateral responses in the contralateral paw region of S1 for spontaneous breathing mice under ketamine–xylazine. Increasing the field strength to 15 T reveals additional pattern in S2 and thalamic regions not visible at 9.4 T. In contrast large bilateral patterns were observed in ventilated mice under 1% isoflurane. **(B)** Signal time courses depict substantial surges in mean arterial blood pressure (MABP) and heart rate (HR) in ventilated, but not in spontaneous breathing animals. Data were acquired in-house reproducing findings from Reimann et al. (2018) and Shim et al. (2018). Functional map superimposed on gray shading (15 T) is adapted with permission from Jung et al. (2019).

While this is in principle an encouraging situation, the reality of implementing mouse fMRI in the laboratory is fraught with peril. Sensory fMRI requires meticulous fine-tuning of murine physiology to compensate for anesthetic side effects, in conjunction with advanced technical equipment to boost the BOLD effect, which increases linearly with the magnetic field strength and venous blood volume (Kim, 2018). A recent study vividly illustrated how the field strength determines whether or not activation of entire brain areas may be detected. Subcutaneous electrostimulation of the murine paw is expected to elicit activity in the contralateral ventral thalamic nuclei, which relay the signal along the spinothalamic tract to the primary (S1) and secondary (S2) somatosensory cortices. Nevertheless, BOLD responses to electrostimulation were found to be limited to the contralateral S1 in mice under ketamine–xylazine at 9.4 T (Figure 5A; Shim et al., 2018), and under medetomidine at 9.4 (Nasrallah et al., 2014a) and 11.7 T (Adamczak et al., 2010). However, the same ketamine–xylazine protocol at 15 T revealed the expected BOLD patterns in all three somatosensory key areas (Figure 5A; Jung et al., 2019).

To boost the BOLD signal, small-bore MR scanners can be equipped with a cryogenically cooled radiofrequency coil that amplifies the temporal SNR by a factor of up to 3 (reviewed in Niendorf et al., 2015). Increasing the statistical power by multiple repetitions of a task similarly facilitates the detection of weak BOLD effects. Both strategies reduce noise, but do not increase the sensitivity to T_2*_—the measure of relative changes in blood oxygenation, which determines the intrinsic technical detection limit of an MR system. To amplify a signal, it must be above the detection threshold. However, weak BOLD responses have been observed in the S2 and the thalamus at 9.4 T (Jung et al., 2019), strongly suggesting that these areas can be detected *via* signal amplification. The studies reported above employed MR surface coils that lose sensitivity for deeper brain areas. In addition to this technical issue, the low BOLD signal in deeper brain areas is likely physiological in nature, and may be caused by the sparser overall thalamic cell density, compared to the cortex (Meyer et al., 2013), by different densities of noradrenergic afferents in these areas (King et al., 1995; Wang et al., 1996) or otherwise asymmetric impact of reduced subthalamic drive, by unequal anesthetic effects on the vasculature in different brain regions (Lei et al., 2001), or by the experimental setup that can create a thermal gradient between the dorsal–ventral axis of the mouse brain (see below).

#### Vasomodulation, Body Temperature, and Respiratory Depression

The modulation of vascular properties across the brain is a key challenge for fMRI under anesthesia—even more so due to the temperature sensitivity of vessels. All anesthetics affect thermoregulation and render mice poikilothermic. Due to their high surface area-to-mass ratio, mice quickly adopt to their environmental temperature. Although blood circulation distributes heat energy across the body, heating pads below the trunk and cool surface MR units attached to the scalp may induce thermal gradients across the mouse, which means the brain temperature is neither homogeneous across all areas, nor accurately reflected by the temperature reading of a rectal probe (Reimann et al., 2016). Aside from a thermal dependency of neural activity that has been shown to alter brain states, and hence anesthetic depth (Volgushev et al., 2000; Reig et al., 2010; Sheroziya and Timofeev, 2015; Schwalm and Easton, 2016), vessels dilate with increasing temperature. Vasoconstriction induced by cooling the scalp can boost cortical BOLD responses to electrostimulation by about 30%, although this is not physiological (Baltes et al., 2011).

Many anesthetics directly affect vascular dilation and reactivity, which can substantially alter baseline CBF and hemodynamic responses (see Table 2, Franceschini et al., 2010; Masamoto and Kanno, 2012). In contrast to cooling, vasoconstrictive effects of α2AR such as medetomidine and xylazine (Sinclair et al., 2003; Fukuda et al., 2013) might hinder the thorough vasodilation of arterioles in response to neural activity and impair BOLD responses in mice (Nasrallah et al., 2014a; Schroeter et al., 2014). Anesthetic-induced vasodilation, on the other hand, increases the baseline blood flow and hence decreases the magnitude of relative changes in CBV, CBF, and BOLD in response to neural activity. Isoflurane is commonly reported to have strong vasodilatory effects (Sharp et al., 2015; Petrinovic et al., 2016; Cao et al., 2017), causing decreases of up to 75% in CBF responses to whisker stimulation at a concentration of only 1% in spontaneously breathing mice (Takuwa et al., 2012). Although isoflurane has been shown to directly dilate cerebral vessels (Iida et al., 1998; Leoni et al., 2011), its major vasodilatory potency is a result of its suppressive effects on respiration in a dose-dependent manner (van Alst et al., 2019).

Respiratory depression leads to hypercapnia—elevated blood CO_2_ levels—which causes strong cerebral vasodilation and increases the baseline CBF (Kety and Schmidt, 1948; Raper and Levasseur, 1971; Shimosegawa et al., 1995; Jones et al., 2005; Leoni et al., 2011; van Alst et al., 2019). Both rats and mice exhibit massively reduced or even fully ablated BOLD responses, when CO_2_ (5–10%) is added to the inspiratory gas (Schlegel, 2017; Munting et al., 2019; van Alst et al., 2019). Mechanically ventilated rats showed substantially higher BOLD responses to subcutaneous electrostimulation, and lower baseline CBF compared to spontaneously breathing rats at the same concentrations of isoflurane (van Alst et al., 2019). A comparison of cortical and thalamic baseline perfusion across anesthetic protocols in mechanically ventilated mice found isoflurane to be in a similar range as urethane, which is known to preserve hemodynamics and vascular diameters (Schroeter et al., 2014).

Hypercapnia has further effects on brain states. Since CO_2_ forms carbonic acid in water, elevated CO_2_ makes the blood more acidic. Both low pH and CO_2_ have been shown to impair neural transmission and excitability (Coulter et al., 1995; Tombaugh and Somjen, 1996; Sun et al., 1997; Meuth et al., 2003, 2006; Putnam et al., 2004; Williams et al., 2007; Sinning and Hübner, 2013). Hyperoxia, on the other hand, has been reported to cause vasoconstriction of arterioles (Duling and Berne, 1970; Pries et al., 1995) and to decrease baseline CBF (Matsuura et al., 2001; Matsuura and Kanno, 2002). This demonstrates that blood gas levels are crucial in fMRI and that respiration should be kept at physiological levels to preserve neural and especially hemodynamic responses.

#### Mechanical Ventilation and Stress Response

This has led to the widespread use of mechanical ventilation in murine fMRI, initially to detect highly reproducible BOLD responses to *salient* (“attention grabbing”) stimuli, like subcutaneous electrostimulation (Bosshard et al., 2010; Baltes et al., 2011), and later nociceptive heat stimuli (see “Nociception, Pain, and Physiological Confounds” section; Bosshard et al., 2015; Reimann et al., 2016). Mechanical ventilation was further introduced as part of a robust protocol for pharmacological fMRI in mice (Ferrari et al., 2012) and is utilized in resting-state fMRI (see “Functional Connectivity and Murine Resting-State fMRI” section; Grandjean et al., 2014). The commonly applied protocol features 1.0–1.3% isoflurane in combination with neuromuscular blockage to minimize motion-related noise and prevent stimulus-correlated movements. An application of classical electrostimulation block paradigms to the murine paw—stimulus trains of 15–30 s composed of rectangular pulses of 0.5–2 mA—revealed that this protocol leads to BOLD signal changes of substantial magnitudes, and a very specific pattern of distribution: BOLD effects occurred in bilateral clusters in S1, S2, the thalamus, and the insular cortex (Figure 5A; Bosshard et al., 2010; Baltes et al., 2011; Schroeter et al., 2017; Reimann et al., 2018; Schlegel et al., 2018; Shim et al., 2018), and were accompanied by strong, transient elevations in mean arterial blood pressure (MABP) and heart rate (HR; Figure 5B; Schroeter et al., 2014; Reimann et al., 2018). This was confirmed for various anesthetic protocols including low-dose isoflurane, medetomidine, propofol, and urethane in ventilated and paralyzed mice (Schroeter et al., 2014).

The same stimulation paradigm applied to spontaneously breathing mice evoked less prominent BOLD responses that were strictly limited to the paw region of the contralateral S1 as described above (Figure 5A; Adamczak et al., 2010; Nasrallah et al., 2014a; Shim et al., 2018). This was observed for medetomidine and ketamine–xylazine anesthesia. The additional patterns observed in contralateral thalamic relay nuclei and S2 at higher field strengths emphasize the somatosensory nature of the response along the spinothalamic tract (Jung et al., 2019). Weak responses in ipsilateral S1 have also been reported for some animals, likely due to inter-hemispheric projections (Schroeter et al., 2017; Shim et al., 2018; Jung et al., 2019), and no cardiovascular changes were observed during the stimulus periods in these studies (Figure 5B).

In contrast, the strong, transient cardiovascular surges in ventilated animals clearly indicate sympathetic activity, which is governed by medullary control areas and triggered by an autonomic stress response (Pfaff, 2005; Samuels and Szabadi, 2008b; Ulrich-Lai and Herman, 2009). Such a response involves activity of the locus coeruleus and other key nuclei of the brainstem and the forebrain arousal system, which innervate multiple cortical and subcortical areas (Figure 1A; Toussay et al., 2013; Lecrux and Hamel, 2016; Lecrux et al., 2019).

The specific reason for this strong sympathetic response in ventilated mice is not fully understood. It is known that endotracheal intubation and forced ventilation can induce excessive stress in humans and animals when the anesthetic depth is too low. We hypothesize that long stimulus trains in ventilated mice accumulate to engage the activity of subcortical arousal nuclei that drive the brain state gradually toward desynchronization (see “Metastability of Brain States, Hysteresis and Behavioral Monitoring” section; Hudetz et al., 2003; Solt et al., 2014; Vazey and Aston-Jones, 2014; Pal et al., 2018; Hayat et al., 2019). Elevated sympathetic activity causes a tightly coupled increase in cardiovascular and respiratory output (Pfaff, 2005; Ulrich-Lai and Herman, 2009). Yet, in ventilated mice, the respiration rate is fixed (to 80–90 bpm), which may lead to an allosteric mismatch (“prediction error”) between autonomic command and sensory feedback (Alheid and McCrimmon, 2008; Kleckner et al., 2017). It is unclear whether this drives the animal’s stress response. However, it appears that ongoing electrostimulation triggers an excitatory feed-forward loop that forces increased activity in reticulo-thalamo-cortical circuits, and causes bilateral patterns in the S1 barrel fields, and strong sympathetic outflow (Toussay et al., 2013; Lecrux and Hamel, 2016; Lecrux et al., 2019).

With isoflurane, this is a transient process, and mice usually fall back into stable sedation after a stimulation period, which is evident from HR and MABP traces (Schroeter et al., 2014; Reimann et al., 2018) as well as from behavioral observations in unparalyzed mice. Mice administered medetomidine while under ventilation have been shown to exhibit increases in their HRs progressively over the length of the fMRI scan following the first stimulation period (Schroeter et al., 2014). Medetomidine exerts its effects predominantly by inhibiting the locus coeruleus (Akeju and Brown, 2017). Once a competing mechanism engages this key arousal nucleus, the sedation is temporarily suppressed (Hayat et al., 2019), and the stimuli might be processed in the awakening, largely unaffected cortex (see “α2AR Agonists Induce a Sleep-Like State by Suppressing General Arousal” section). Again, medetomidine produces a state from which arousal is more likely to occur than through the use of GABAergic drugs, which suppress the activity of excitatory neurons across the entire brain (Figures 1B,C).

Mechanically ventilated mice anesthetized with medetomidine exhibited bilateral BOLD responses even to single electrical pulses applied to the paw (1 mA, 0.5 ms; Schlegel et al., 2015). The same single electrical pulses applied to ventilated mice under isoflurane, propofol, or urethane evoked BOLD responses contralaterally along the spinothalamic tract in S1, S2, and first-order TC nuclei at 9.4 T—very similar to the patterns observed for stimulus trains in spontaneously breathing mice under ketamine–xylazine anesthesia at 15.2 T (Figure 5; Jung et al., 2019), with the latter being more spatially defined. The greatest similarities have been reported for patterns elicited under propofol along with profound hemodynamic responses to electrostimulation. However, the sedation under propofol was found to be unstable for the adjusted dosage (Schroeter et al., 2014; Schlegel et al., 2015). In summary, for the tested regimens, single pulses of electrical stimulation in mechanically ventilated mice elicit patterns predominantly along the somatosensory axis, whereas long pulse trains evoke bilateral patterns that likely reflect a transient recruitment of arousal structures, which can drive the brain state towards consciousness.

Multiple publications have reported on subcutaneous electrostimulation of the paw in mechanically ventilated mice under isoflurane. Whether this protocol allows for the detection of non-salient, low-intensity sensory stimuli has not yet been investigated. There is evidence that stimulus trains in the somatosensory range (0.5 mA) lead either to bilateral patterns or to no response at all (Shim et al., 2018). For a free-breathing sedation adjusted at 0.5–1% isoflurane, significant BOLD responses to whisker deflection have been reported in a murine fMRI study (Kahn et al., 2011). In such lightly sedated mice, tracheal intubation and forced ventilation would act as heavy continuous stressors during an fMRI experiment. Accordingly, mechanical ventilation does not permit further decreases in isoflurane concentrations, and approximately 1–1.3% are advised to prevent panic and withdrawal from the endotracheal tube. Concentrations of 0.75–1.1% isoflurane were found necessary to avoid burst suppression and permit stable sedation in spontaneous breathing mice (Kozai et al., 2015; Michelson and Kozai, 2018). To create conditions as close as possible to those experienced by a calm human volunteer, alternative strategies to ventilation should be considered for sensory tasks.

#### Balancing Anesthesia in Spontaneously Breathing Mice

Increasing oxygen concentration in the carrier gas is a popular strategy to prevent hypocapnia in spontaneously breathing mice, although caution is advised: no differences in respiratory rates, pH, blood CO_2_ levels, or blood pressure have been found for mice breathing isoflurane in pure oxygen (100%) compared to medical air (21%). This has been explained by a decrease in lung volume for pure oxygen based on alveolar collapse, known as *absorption atelectasis* (Wilding et al., 2017). Hemodynamic responses to whisker stimulation were significantly reduced for mice breathing pure oxygen compared to medical air (Sharp et al., 2015). The exact concentration of oxygen in the breathing gas may be adjusted based on a bell-shaped stimulus–response curve with respect to the anesthetic protocol that is applied (Blazquez Freches et al., 2018).

A good strategy is to apply anesthesia that causes neither respiratory depression nor vasodilation. This makes α2AR agonists attractive to assess sensory processing in murine fMRI, preserving physiological breathing rates at about 120–190 bpm (Adamczak et al., 2010; Nasrallah et al., 2014a). However, α2AR agonists induce a sleep-like state and impair BOLD responses due to vasoconstriction (see above; Fukuda et al., 2013; Nasrallah et al., 2014a), which further initiates a transient increase in MABP followed by a reduction based on suppression of noradrenergic sympathetic ganglia (McCallum et al., 1998; Samuels and Szabadi, 2008a). A better choice might be the combination of two or more complementary anesthetics, to balance their respective actions and to tailor the desired effect (Fukuda et al., 2013). Combining the α2AR agonist xylazine together with ketamine preserves physiological breathing rates at about 180 bpm and leads to pronounced BOLD responses, at least in the contralateral S1 (Shim et al., 2018). Another promising alternative was introduced by balancing isoflurane anesthesia with fentanyl-fluanisone and midazolam, which allows the isoflurane level to be reduced to 0.5–0.8% (Sharp et al., 2015). This was reported to induce stable sedation and permit the detection of hemodynamic effects for whisker stimulation, with magnitudes and transition times similar to those observed in awake mice.

Balanced multimodal anesthesia holds great promise to circumvent the multiple challenges in murine fMRI, with the aim of preserving physiological conditions while sufficiently sedating the animal, and still ensuring a certain level of “connectedness” to sensory perception and interoception (see “Anesthetic Depth and Consciousness–the Virtue of Translational Neuroimaging” section; Sanders et al., 2012). Protocols for balanced multimodal anesthesia will be further discussed in “Multimodal Anesthesia in Translational fMRI” section.

### Nociception, Pain, and Physiological Confounds

Nociception is “the neural process of encoding noxious stimuli.” Pain is defined as an unpleasant *experience* that may or may not arise from nociception (Davis et al., 2017). In contrast to fMRI tasks that probe the central processing of applied stimuli, fMRI of pain explicitly aims to identify neural correlates of a phenomenal quality (Mouraux and Iannetti, 2018). Its assessment is therefore hampered in the (sedated) rodent, which cannot report on the experience or level of pain during an fMRI task (Seth et al., 2005). This is an important domain for translational fMRI: in principle, it should be possible to first correlate nociceptive-evoked BOLD patterns with subjective ratings of pain to identify pain-specific functional neurosignatures in the human brain (Wager et al., 2013; Woo et al., 2017). In a second step, a corresponding signature could be sought in the sedated animal model (Tracey, 2017). This leads to an even more fundamental question in terms of anesthesia: to what extent is consciousness required to encode a pain-specific neurosignature?

Currently, we cannot answer this question, since a pain-specific neurosignature has not yet been found (Mouraux and Iannetti, 2018). Painful stimuli are processed within a widely distributed network that is often referred to as the “pain matrix” (Legrain et al., 2011)—a term derived from Melzack’s original *neuromatrix theory of pain* (see below; Melzack, 1989, 2005). It has been shown that salient and painful stimuli are processed by largely overlapping cortical areas in humans (Mouraux et al., 2011), and subcortical and arousal structures that are involved in nociception are also engaged by acute responses to stress (Reimann et al., 2016; Martins and Tavares, 2017). In other words, the neuromatrix is not pain-specific. This is a major obstacle for identifying pain-specific brain signatures, because painful stimuli are: (1) intrinsically salient (Legrain et al., 2011; Mouraux et al., 2011); and (2) often provoke autonomic stress responses (Reimann et al., 2016, 2018).

**Figure 6:**
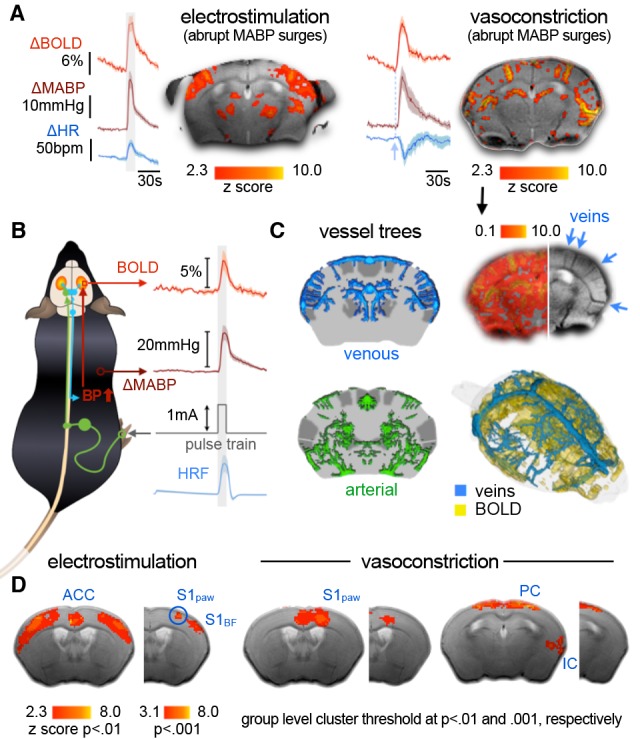
BOLD patterns evoked by abrupt surges in MABP.** (A)** Electro-stimulation evokes abrupt surges in MABP and HR. Mimicking these surges by intravenous injection of vasoconstricting drugs leads to BOLD patterns co-locating with large veins. Injection time point is indicated by dashed line. **(B)** Nociceptive stimuli or stress can elicit abrupt surges in MABP that increase the influx of oxygenated blood into the brain vasculature. MABP and BOLD patterns are highly correlated with each other and with the applied stimulus. This causes the hemodynamic response function (HRF)—that is modeled based on the stimulus paradigm—to reveal MABP-induced effects as significant patterns. **(C)** Significant patterns co-localize with large veins and can therefore be identified and corrected for, whereas widespread patterns remain below the statistical threshold. **(D)** At the group level, significant effects persist at more liberal thresholds, and few minor clusters even at more conservative standards. Adapted with permission from Reimann et al. (2018).

Such stress responses introduce severe complications in murine fMRI. They involve the activity of the nociceptive–medullary–autonomic circuit—a fundamental component of the “flight or fight” response, which triggers abrupt surges in HR and MABP (Figure 6A; Price, 2000; Pfaff, 2005; Brown et al., 2010). Since cerebral autoregulation has high-pass filter characteristics, slow changes in MABP are buffered to a certain extent, whereas more pronounced and rapid changes are reflected in the CBF (Hamner et al., 2019). The efficiency of dynamic autoregulation might be further impaired by vasomodulatory effects of anesthesia (Lee et al., 1994; Dagal and Lam, 2009; Sanders et al., 2011). Abrupt surges in MABP, well within the physiological range (80–120 mmHg in mice), can lead to stimulus-correlated increases in CBF causing widespread BOLD patterns across the brain (Figures 6B,C; Kalisch et al., 2001; Wang et al., 2006; Gozzi et al., 2007; Reimann et al., 2018). Veins are particularly prone to translate changes in CBF, into pronounced BOLD effects due to their low oxygen concentration (Reimann et al., 2018). In fact, significant clusters were observed along large draining veins (cluster threshold *z* = 3.1) that were highly correlated with transient MABP surges induced by methods of controlled pharmacological vasoconstriction (Figures 6A,D). Since large veins co-localize with key cortical regions of the neuromatrix (S1, S2, insula, and parietal cortex), this is a confounding factor in current murine fMRI studies of nociception. Aside from strategies to identify and correct the affected regions, fMRI protocols that track changes in CBV, such as vascular space occupancy (VASO) or iron-oxide-based CBV-fMRI, are less prone to such large-vein effects (for a review, see Huber et al., 2017) and can be used either as alternative techniques, or to normalize and correct BOLD data.

Further complexity is introduced by outreaching vasodilatory projections of specific arousal nuclei such as the locus coeruleus, or the nucleus basalis of Meynert in the basal forebrain that are thought to have a substantial impact on the cortical hemodynamic responses to noxious stimuli (Lecrux and Hamel, 2016; Uchida and Kagitani, 2018; Paquette et al., 2019). The extent to which these BOLD responses actually reflect neural activity remains unclear (Lecrux et al., 2019). Both abrupt elevations in MABP and remote vasodilatory projections of arousal nuclei can produce hemodynamic readouts that are indistinguishable from neurovascular coupling. This problem has hardly been explored and is currently considered a major hurdle for interpreting experimental data related to nociceptive fMRI in (ventilated) rodents (Paquette et al., 2018).

**Figure 7:**
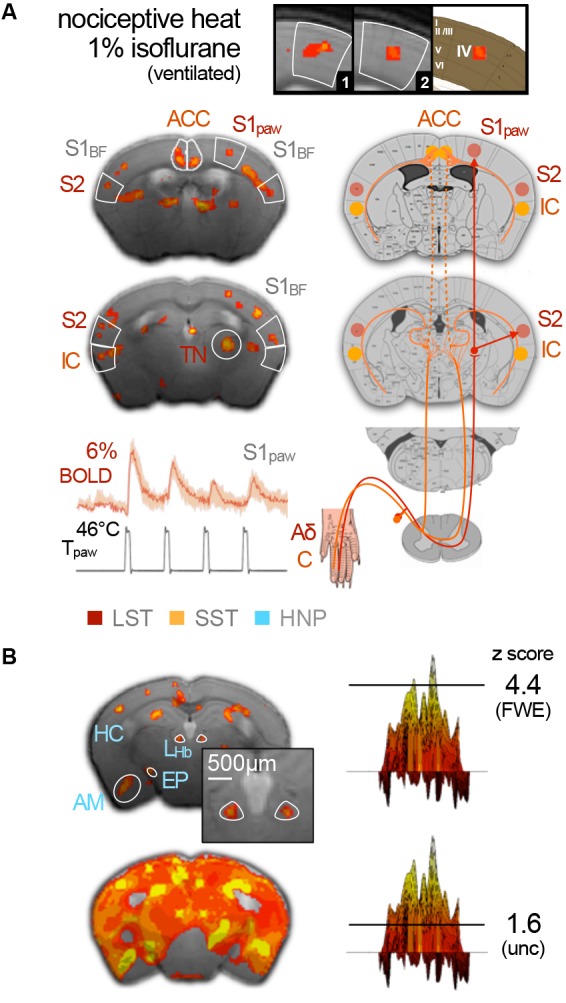
Murine nociception induced by heat stimuli applied to the paw.** (A)** BOLD patterns along the spinothalamic (LST) and spinoreticular tract (SST) depict well-known nociceptive routes in the mouse with high spatial accuracy, including thalamic nuclei (TN), primary (S1) and secondary (S2) somatosensory cortex, the barrel field (S1_BF_), and insular (IC) and ACC. **(B)** Involvement of the habenula nociceptive pathway (HNP) has also been observed, including amygdala (AM), hippocampal areas (HC), entopenduncular nucleus (EP), and the small nuclei of the lateral habenula (L_Hb_). These highly precise patterns represented the tip of the iceberg of underlying BOLD effects, likely induced by MABP surges and vasodilative projections from subcortical arousal nuclei. Adapted from Reimann et al. (2016), http://creativecommons.org/licenses/by/4.0.

Despite these considerations, nociceptive pathways are largely preserved in murine BOLD fMRI using low-dose mono-anesthetic isoflurane anesthesia in mechanically ventilated and paralyzed mice (Reimann et al., 2016). Brief heat stimuli just above the Aδ nociceptor threshold (46°C) applied to the murine paw evoked BOLD effects in brain areas including the spinothalamic and spinoreticular tract, which have been well documented as having a functional involvement in murine and human nociception (Figure 7A). Employing a cryogenic transmit-receive unit (Niendorf et al., 2015) at 9.4 T has permitted resolving even the small nuclei of the habenular nociceptive pathway (Figure 7B, Reimann et al., 2016)—a pain modulating circuit known from human fMRI and from histological c-fos immunostaining in the mouse (Shelton et al., 2012a, b).

The detail of the BOLD patterns observed in this study was achieved by highly conservative thresholding, and has exposed peak values of extensive patterns spreading across large parts of the brain (Figure 7B). More liberal thresholding of heat-evoked BOLD effects in mice under comparable conditions revealed pattern distributions very similar to those observed for electrostimulation (Bosshard et al., 2015). In fact, nociceptive and arousal pathways are tightly interconnected (Craig, 2013). It is thus difficult to disentangle or exclude a potential contribution of ventilation-driven stress responses from those involved in nociception (as discussed in “Sensory Processing and the Key Challenges in Murine fMRI” section). Nociception drives arousal pathway activity, and hence cortical desynchronization, toward a transient emergence from anesthesia (Figure 2E; Hudetz et al., 2003; Solt et al., 2014; Vazey and Aston-Jones, 2014; Akeju and Brown, 2017; Sanders, 2017; Hayat et al., 2019). Therefore, it is important to develop protocols that permit stable sedation in receptive, spontaneously breathing animals. The continuous monitoring of MABP permits the tracking of this important confounder, but it is highly invasive and introduces postoperative nociception. Sympathetic activity is similarly reflected in HR and respiration. Both may rise with the stimulus, but should subsequently return to the baseline (Reimann et al., 2016, 2018). A progressively rising HR requires adjustment of the anesthetic dosage to avoid the animal waking (Nasrallah et al., 2014b; Schroeter et al., 2014; Shim et al., 2018).

The interconnection of nociceptive and arousal routes causes a transient lightening of anesthesia in response to nociceptive stimuli. This could explain why BOLD responses to noxious electrostimulation are partially preserved in humans even after propofol-induced LOC, whereas sensory evoked patterns vanish (see “Anesthetic Depth and Consciousness–the Virtue of Translational Neuroimaging” section; Lichtner et al., 2018). In relation to our initial question, of whether consciousness is required to evoke a pain-specific neurosignature, it could be said that nociception itself drives the brain state toward consciousness. The proposed architecture of the *body-self neuromatrix* is composed of sensory, affective, and cognitive subnetworks of brain areas. Their output patterns produce the multiple dimensions of the pain experience (sensory-discriminative, cognitive-evaluative, motivational-affective), which coincide with homeostatic and behavioral responses (Melzack, 2005; Craig, 2013; Seth and Tsakiris, 2018). Dynamic changes in the FC of these respective networks, triggered either by external nociceptive stimuli or by internal processes in animal models of chronic pain, may offer a more concrete representation of the neuromatrix, as was recently addressed as the dynamic pain connectome (Kucyi and Davis, 2017).

### Functional Connectivity and Murine Resting-State fMRI

**Figure 8:**
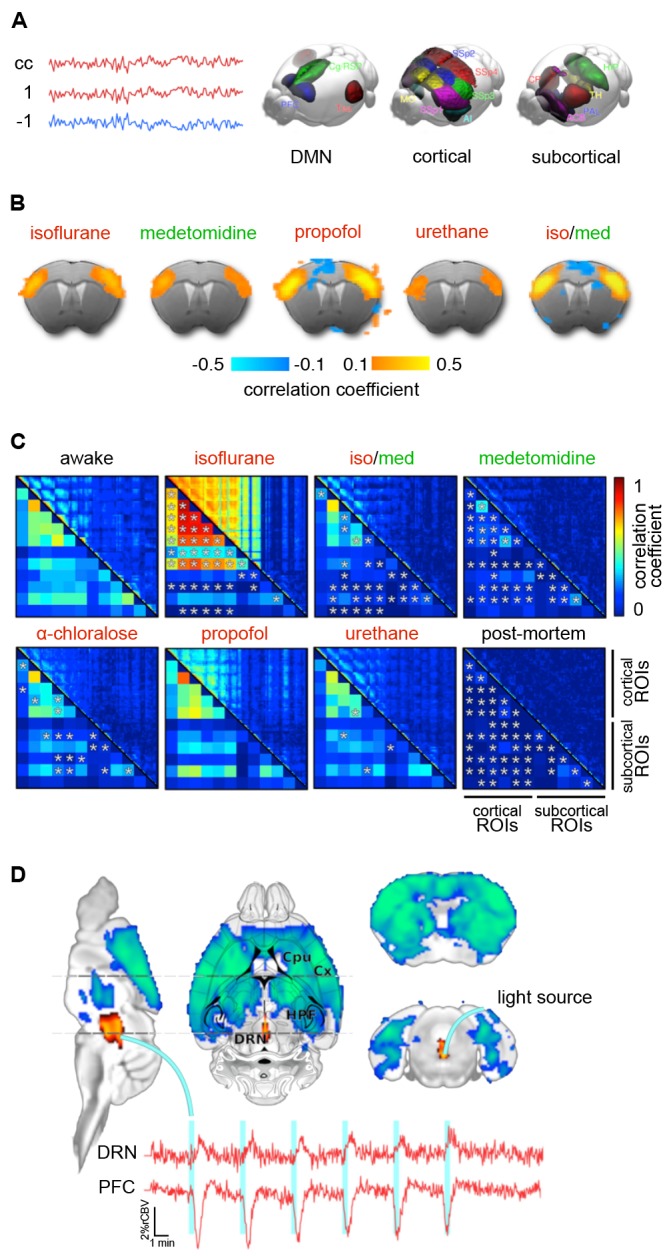
Functional connectivity (FC) in the mouse brain. **(A)** FC is defined by the correlation coefficient (cc) of remote brain areas. The left panel shows an assembly of important areas in murine FC, including the default mode network (DMN), cortical sensory-motor networks, and subcortical networks. **(B)** Murine FC correlation maps for five anesthetic protocols. Seed voxels in the anterior primary somatosensory cortex show anticorrelated time courses in the cingulate cortex. Anticorrelation is an essential criterion for anesthetic states in fMRI in which the FC is not restricted to its structural scaffold. **(C)** Correlation matrices in the rat for six different anesthetic protocols referenced against awake rats. Asterisks indicate statistically significant differences compared with the awake group (*t*-test, *p* < 0.05, false discovery rate corrected). **(D)** Optogenetic stimulation of the dorsal raphé nuclei that send serotinergic projections into large parts of the brain leading to wide patterns of negative BOLD effects. Adapted from **(A)** Grandjean et al. (2019a); **(B)** based on Grandjean et al. (2014), reproducible using original data from the open repository, https://central.xnat.org; **(C)** adapted with permission from Paasonen et al. (2018), **(D)** Grandjean et al. (2019b).

FC between two or more brain areas is defined by the correlation coefficient of their signal intensity courses over time: the higher the correlation, the higher the FC (Figure 8A; Petersen and Sporns, 2015). This simple relation is the key principle in defining functional networks across the entire brain—an opportunity that is unique to fMRI. FC is considered to be in large part the hemodynamic translation of simultaneous activity of neural populations, which is inherently limited by the physical connections between neurons (Mateo et al., 2017). This structural scaffold permits a repertoire of possible functional configurations that changes dynamically on a time scale depending on the level of organization: on the order of 1 ms for neurons, 10 ms for local circuits, 100 ms for EEG, and 1 s for fMRI (Mashour and Hudetz, 2018). This has two important implications: (1) the temporal changes in FC hold important information that is simply discarded when large fMRI time series are averaged assuming steady-state conditions (Preti et al., 2017); and (2) brain states can, in principle, be translated into FC and assessed *via* fMRI (Van de Ville et al., 2010).

The integrity or disruption of FC is commonly used as an indicator of various types and stages of neurodegenerative diseases and neurophysiological malfunctions, or to study the effects of pharmacological interventions, genetically modified proteins or neural populations under optogenetic control (see “Deep Brain Stimulation, Opto- and Chemogenetics” section). Notably, the majority of murine resting-state studies have been conducted under anesthesia (Jonckers et al., 2011, 2014, 2015; Guilfoyle et al., 2013; Grandjean et al., 2014; Mechling et al., 2014; Nasrallah et al., 2014a; Sforazzini et al., 2014; Liska et al., 2015; Bukhari et al., 2017; Wu et al., 2017; Pan et al., 2018). The extent to which various anesthetic protocols intrinsically disrupt FC is therefore a critical issue that must be comprehensively addressed. A number of excellent reviews are already available on this issue for humans (Mashour and Hudetz, 2017, 2018) and mice (Hoyer et al., 2014; Jonckers et al., 2014, 2015; Pan et al., 2015; Gozzi and Schwarz, 2016; Chuang and Nasrallah, 2017; Sumiyoshi et al., 2019; Grandjean et al., 2020; Mandino et al., 2020). Here, we add to the existing literature by focusing on basic concepts of anesthesia-specific changes in FC with respect to the underlying mechanisms.

FC is remarkably similar across mammalian species. In 2014, 3 years after the first study on murine FC (Jonckers et al., 2011), it was shown that mice exhibit a medial fronto-parietal or “default mode” network (DMN) during the resting state (Stafford et al., 2014) similar to that of rats (Lu et al., 2012), monkeys (Vincent et al., 2007), and humans (Raichle et al., 2001). The DMN plays a fundamental role in baseline functions of the mammalian brain (Raichle, 2015). In humans, it is associated with mind-wandering and rumination. Whether the DMN entails comparable phenomenal qualities in mice remains speculative and may be interesting from an evolutionary or philosophical point of view (Seth et al., 2005; Havlik, 2017). In the presence of a salient stimulus, DMN connectivity becomes disrupted and shifts towards connectivity in the dorsal attention network (Raichle, 2015), in the same way that cortical states change with attention toward a stimulus (see “Cortical States of Wakefulness” and “Brain States Under Anesthesia” sections).

The DMN involves similar higher-order cortical regions along the medial fronto-parietal axis across species (Figure 8A; Raichle, 2015; Grandjean et al., 2020), although prefrontal and cingulate regions appear to perform the tasks of the precuneus, which is absent in rodents (as reviewed in Gozzi and Schwarz, 2016). Further functional networks have been described in the mouse, including a salience network and latero-cortical network, as well as various sensory, sensorimotor, cerebellar, limbic system, and basal ganglia networks (see Figure 8B for murine FC maps based on a seed voxel in S1 for different anesthetics; Jonckers et al., 2011, 2014; Grandjean et al., 2014, 2017b, 2020; Nasrallah et al., 2014b; Sforazzini et al., 2014; Zerbi et al., 2015; Shim et al., 2018; Gutierrez-Barragan et al., 2018).

#### Anesthesia-Induced Disruptions in FC

The network architecture is very similar between humans and mice (Bullmore and Sporns, 2012; Gămănuţ et al., 2018; Balsters et al., 2019; Fulcher et al., 2019), which has been utilized to pursue questions about human network configurations in the mouse (Fulcher and Fornito, 2016). Brain networks are organized around highly connected hubs. Substantial disruption of FC induced by anesthesia at these central nodes can cause widespread communication failure associated with LOC (Schröter et al., 2012; Lee H. et al., 2013; Moon et al., 2015; Bonhomme et al., 2016; Tononi et al., 2016; Mashour and Hudetz, 2018). Abnormal reorganization of hubs during recovery from anesthesia likely explains differences in FC strength and configuration observed prior to and following LOC (Långsjö et al., 2012; Liu et al., 2013b; Monti et al., 2013). This sheds light on the anesthetic hysteresis effect: namely, that different anesthetic dosages are required to induce a similar depth of anesthesia during the induction and recovery phases (see “Brain States Under Anesthesia” section). The translational utility of murine FC studies may depend on the capacity of the anesthetic protocol to preserve or restore the integrity of specific functional networks across the brain.

Of course, the technical equipment, animal preparation protocols, arousal states due to stress, murine susceptibility to vascular effects of anesthesia, and the applied statistical methods all have a crucial impact on the reliability of the data (Grandjean et al., 2020). It is therefore helpful to reference murine FC data against: (1) FC data derived from more stable systems like humans, monkeys, or rats under the same anesthesia; and (2) the molecular pathways that are known to be targeted by specific anesthetic protocols. Disruption of neural communication across brain areas occurs according to the principal target routes of an anesthetic regimen (see “Brain States Under Anesthesia” section) and may be translated into a discrete FC signature (Table 3). This relation makes the assessment of FC a highly relevant point of convergence for detailing and understanding brain states induced by anesthesia.

**Table 3:** Functional connectivity (FC) disruptions reflect principal mechanisms of anesthesia.

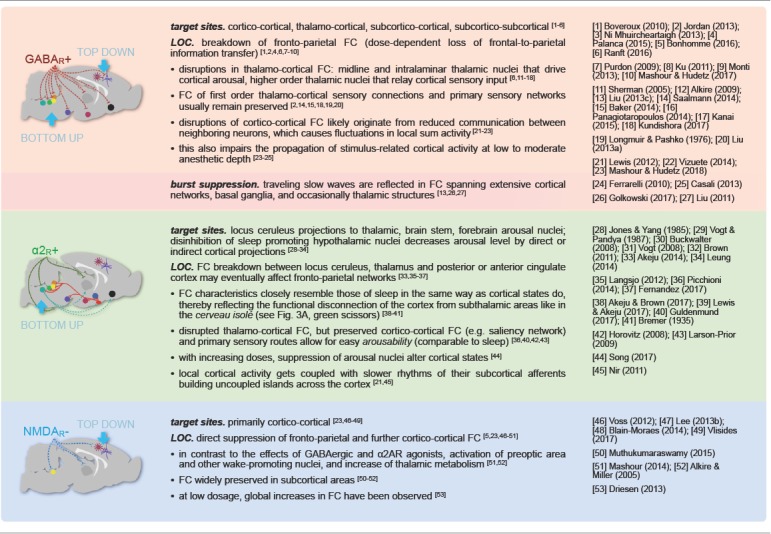

*The table summarizes key effects on FC for the three major anesthetic classes: disruption of whole brain FC by GABA_A_ receptor agonists (GABA_R_+, red), bottom-up disruption by α2 adrenoreceptor agonists (α2_R_+, green), and disruption of cortical FC by NMDA receptor antagonists (NMDA_R-_, blue). Loss of consciousness (LOC) is most likely mediated by suppression of frontal-to-parietal information transfer in the cortex and can be achieved by all three anesthetic classes either directly *via* disruption of cortico-cortical FC or indirectly *via* bottom-up pathways. Principal mechanisms of anesthesia are described in “Brain States Under Anesthesia” section and their reflection in FC patterns is discussed in “Functional Connectivity and Murine Resting-State fMRI” section. References are shortened to first author (and year) to save space and allow for comprehensive referencing*.

#### Anticorrelation and Rich Dynamic Repertoires—Criteria for Appropriate FC

Discrete signatures of GABAergic drugs and volatile ethers, which suppress brain-wide neural activity include dose-dependent disruption of FC across the entire brain (for details, see Table 3; Boveroux et al., 2010; Schröter et al., 2012; Jordan et al., 2013; Ní Mhuircheartaigh et al., 2013; Palanca et al., 2015; Bonhomme et al., 2016; Ranft et al., 2016). This is reflected in a reduction in global connectedness, with stronger effects on higher-order cortical and thalamic FC compared to sensory areas, and fewer transitions between functional networks (Uhrig et al., 2018; Golkowski et al., 2019). This rigidity in the dynamics between networks correlates with a reduced ability to interact with external stimuli (see “GABAA Agonists Suppress Neural Activity Across the Central Nervous System” section). LOC has consistently been found to be correlated with a breakdown of fronto-parietal FC in humans and non-human primates induced by GABAergic drugs (Purdon et al., 2009; Boveroux et al., 2010; Jordan et al., 2013; Monti et al., 2013; Palanca et al., 2015; Ranft et al., 2016; Mashour and Hudetz, 2017; Uhrig et al., 2018), likely due to impaired frontal-to-parietal cortical information transfer (Ku et al., 2011; Jordan et al., 2013; Ranft et al., 2016). Although this relationship is also thought to exist in mice, further studies are required to assess the modes of murine fronto-parietal FC and their significance for wakefulness (Imas et al., 2005b; Mashour and Alkire, 2013; Sforazzini et al., 2014; Pal et al., 2018; Shofty et al., 2019; Grandjean et al., 2020).

On the other hand, burst suppression and slow waves that propagate unabated across the cortical surface (see “Crossing the Borders: GABAergic Slow Waves and Burst Suppression” section) promote FC that can span extensive cortical networks in humans and rats, occasionally reaching into the basal ganglia and thalamic structures (Liu et al., 2011, 2013b, c; Golkowski et al., 2017; Schwalm et al., 2017; Aedo-Jury et al., 2019). Isoflurane concentrations as low as 1.3% in ventilated rats were found sufficient to induce highly synchronized large-scale FC patterns in fronto-cortical and striatal regions, and to suppress most thalamo-cortical and intra-subcortical connections (Figure 8C; Paasonen et al., 2018).

Loss of information transfer and integration is further reflected in a reduced number of possible FC configurations (Hutchison et al., 2014; Barttfeld et al., 2015; Hudetz and Mashour, 2016; Ma et al., 2017; Uhrig et al., 2018). The repertoire of FC configurations can be evaluated *via* a sliding-window approach. In this approach, a window length of about 1 min is defined and shifted along the entire length of the resting-state time series, with a step size equal to the fMRI temporal resolution (typically 1–2 s). Each time-shifted segment then undergoes a correlation analysis, which gives access to dynamic aspects of FC, which have been reported to be well conserved across rats, monkeys, and humans (Majeed et al., 2011). Although blurred in temporal resolution, this technique permits the assessment of dynamic whole-brain signatures based on FC. Combining Ca^2+^ imaging or electrophysiology with fMRI (Thompson et al., 2013; Keilholz, 2014; Schmid et al., 2016; Schwalm et al., 2017; Lurie et al., 2018; Schlegel et al., 2018; Matsui et al., 2018; Aedo-Jury et al., 2019; van Alst et al., 2019), these signatures can be linked to the cortical states, and hence be employed to determine the nature and level of sedation (see “Brain States Under Anesthesia” and “Limitations in Maintaining Stable Brain States” sections).

The dynamical elaboration of a rich, flexible repertoire in the awake brain becomes increasingly constrained with increasing anesthetic dosage (Barttfeld et al., 2015; Uhrig et al., 2018). This is accompanied by a lack of anticorrelated areas—a further symptom of inflexible FC and confined information traffic pointing to LOC. A lack of anticorrelated areas was reported in mice ventilated at 1% isoflurane (Figure 8B; Grandjean et al., 2014). At 1.3% isoflurane broad cortical FC has been observed along with synchronized cortical slow wave activity in the Ca^2+^ signal assessed in the murine S1 (Schlegel et al., 2018). Further reducing the isoflurane concentration below 1–1.3% is not appropriate if the mice are ventilated and paralyzed (see “Sensory Processing and the Key Challenges in Murine fMRI” section). As head movements, including rhythmic, respiration-induced motion, account for a large part of the noise in FC (Kalthoff et al., 2011; Pais-Roldán et al., 2018), a trade-off has to be made between data quality and anesthetic depth. In ventilated rats, a reduction of isoflurane from 1.5% to 1% was sufficient to preserve distinct cortical networks and anticorrelated FC at a largely desynchronized frequency band in the EEG (Liu et al., 2013c). Due to the narrow dose range between stable sedation and burst suppression, careful adjustment of isoflurane concentrations is required to avoid unphysiological FC.

Compared to isoflurane and other volatile ethers, halothane exhibits a remarkably wide dose range between stable sedation and burst suppression (Murrell et al., 2008; Brown et al., 2018; McIlhone et al., 2018). A rich repertoire of dynamic FC patterns was observed for halothane-anesthetized mice, based on a varied set of recurring configurations of FC, with abundant anticorrelated areas (Sforazzini et al., 2014; Gutierrez-Barragan et al., 2018). At low dosages, other GABAergic drugs have been shown to preserve FC quite well with respect to the above criteria. The use of low-dose propofol and urethane was reported to achieve the best resemblance to FC in awake rats out of six different anesthetic regimens tested (Figure 8C; Paasonen et al., 2018). A similar observation was reported for mice, with propofol preserving spatially defined symmetrical cortico-cortical FC with anticorrelated cingulate areas (Figure 8B; Grandjean et al., 2014). Nevertheless, unstable sedation has been reported for this protocol in mice, which could be due to ventilation conditions, considering the general arousability of mice relative to rats. Comparing the capacity of anesthetic protocols to approximate the FC of the awake state in rats, propofol and urethane showed the best performance, followed by α-chloralose, while a protocol combining isoflurane and medetomidine (see below) was ranked 4th (Figure 8C).

#### “One-Dimensional” Disruption of FC

Medetomidine and other α2AR agonists, which suppress subcortical arousal nuclei (see “Brain States Under Anesthesia” section) disrupt FC in a bottom-up manner (Table 3). Disinhibition of sleep-promoting cells in the hypothalamus disrupts subcortico-subcortical and hence thalamo-cortical FC. This causes a discrete FC signature that reflects the functional disconnection of the cortex from subcortical areas, as anticipated on the basis of the *cerveau isolé* (“Brain States Under Anesthesia” section; Bremer, 1935; Guldenmund et al., 2017; Lewis and Akeju, 2017; Akeju and Brown, 2017). With increasing dosages, local cortical activity gets coupled with slower rhythms of subcortical afferents from the suppressed arousal nuclei, building uncoupled islands across the cortex (Nir et al., 2011; Lewis et al., 2012). This is reflected in disrupted interhemispheric cortico-cortical FC, which has been observed with increasing dosages of medetomidine in rats (Nasrallah et al., 2014a) and mice (Nasrallah et al., 2014b). The mechanism of bottom-up-induced LOC likely involves a breakdown of FC between the locus ceruleus, the thalamus, and the posterior or anterior cingulate cortex, which may eventually affect fronto-parietal FC (Långsjö et al., 2012; Fernandez et al., 2017). Medetomidine was found to preserve moderate intercortical connectivity in ventilated rats, although this was significantly reduced compared to that of awake rats, whereas thalamo-cortical activity was almost absent (Figure 8C; Pawela et al., 2008; Zhao et al., 2008; Paasonen et al., 2018).

Ketamine, on the other hand, which acts primarily in a top-down manner in the cortex, mediates LOC by direct suppression of fronto-parietal and further cortico-cortical FC (Table 3; Voss et al., 2012; Lee U. et al., 2013; Blain-Moraes et al., 2014; Muthukumaraswamy et al., 2015; Bonhomme et al., 2016; Vlisides et al., 2017; Mashour and Hudetz, 2018; Uhrig et al., 2018). In contrast to GABAergic and α2AR agonists, ketamine has been reported to largely preserve subthalamic FC, which is consistent with its activating effects on the preoptic area and other wake-promoting nuclei (Alkire and Miller, 2005; Mashour, 2014). The top-down and bottom-up actions of ketamine and medetomidine may be thought of as “one-dimensional” (Mashour and Hudetz, 2017). The dual effect of GABAergic drugs makes them more suitable for stable sedation by suppressing both subthalamo-cortical and cortico-cortical FC (Boveroux et al., 2010). To achieve a similar anesthetic stability by not overly stressing one pathway, drugs targeting either of these “one-dimensional” mechanisms are commonly combined (e.g., ketamine-xylazine or medetomidine-isoflurane) in order to target the complementary pathways.

#### Multimodal Anesthesia in Murine Resting-State fMRI

Combining isoflurane and medetomidine at levels roughly half their mono-anesthetic dosages (Lu et al., 2012; Paasonen et al., 2018; Sumiyoshi et al., 2019) has been shown to produce FC resembling that of awake rats substantially better than either mono-anesthetic protocol alone, yielding a correlation matrix that roughly corresponds to that of α-chloralose (Figure 8C; Paasonen et al., 2018). This medetomidine-isoflurane protocol was adapted for murine resting-state fMRI, where it was also described as providing the “best of both worlds”: reproducible, spatially defined networks, including symmetrical patterns of FC in bilateral sensory areas anticorrelated with cingulate regions, at a stable sedation in ventilated mice (Figure 8B; Grandjean et al., 2014).

Later studies revealed a high correspondence between FC and structural connectivity of cortico-cortical and cortico-striatal regions with the same medetomidine-isoflurane protocol in mice, whereas thalamo-frontal cortical FC was found suppressed (Grandjean et al., 2017b). Although a high correspondence between functional and structural connectivity has been associated with a constrained repertoire of FC configurations (Barttfeld et al., 2015; Uhrig et al., 2018), the sliding-window approach ultimately revealed rich patterns of dynamic FC under this protocol (Grandjean et al., 2017a). This incongruity could be due to methodological differences between the studies (e.g., the use of MR diffusion-based vs. neural tracer-based structural connectivity; Straathof et al., 2019). The exact relationship between the dynamics of FC and its correspondence to its scaffold as a function of anesthetic depth remains to be clarified across anesthetic protocols in mice.

Taken together, the preservation of a rich repertoire of FC configurations, along with spatially defined and anticorrelated FC in relevant networks, has made medetomidine-isoflurane the protocol of choice for studying murine FC. Low-dose halothane performs similarly well in mice, with the advantage of preserving thalamo-frontal cortical FC (Gutierrez-Barragan et al., 2018; Bertero et al., 2018; Mandino et al., 2020). Halothane is rarely used due to a risk of causing liver damage, although the risk is possibly no higher than that of other halogenated vapors (Mizobe, 2019).

A recent meta-analysis comparison across numerous labs, MR scanners, and animal preparation protocols (Grandjean et al., 2020) revealed greater specificity within elements of the DMN under medetomidine-isoflurane anesthesia in ventilated mice than in awake habituated mice, although the latter exhibited higher overall FC. This can be interpreted as an effect of environmental or internal distraction in awake mice, since DMN connectivity is known to be disrupted in the presence of salient stimuli (see above; Raichle, 2015). Contradictory findings have been reported for anesthetic effects on the DMN, although it appears that FC is principally supported at low anesthesia and disrupted with increasing anesthetic depth (Akeju et al., 2014a; Huang et al., 2014; Palanca et al., 2015; Ranft et al., 2016).

#### Resting-State fMRI in the Awake Mouse

Medetomidine-isoflurane and low-dose halothane are currently considered the most suitable anesthetic protocols available for the assessment of murine FC. Yet, carefully titrated multimodal protocols specifically designed to balance the complementary effects of their anesthetic compounds offer far more room for improvement (see “Multimodal Anesthesia in Translational fMRI” section). Comparing FC matrices for medetomidine-isoflurane against propofol with reference to the “ground truth” of an awake habituated rat gives an immediate impression of what we are heading for (Figure 8C). With respect to the *non-voluntary task conundrum*, resting-state applications have the best chance to produce reliable data from awake habituated mice. Nevertheless, training awake mice to tolerate the noisy scanner environment is far more challenging than for awake rats (Jonckers et al., 2014, 2015; Low et al., 2016b; Dopfel and Zhang, 2018), although further strategies are constantly being developed to facilitate this procedure (Yoshida et al., 2016; Madularu et al., 2017; Han et al., 2019; Chen et al., 2020).

Since brain states during wakefulness alter with the level of arousal, such a “ground truth” is still prone to reflect stress and restraint rather than the resting state, which becomes critical when comparing results with human volunteers (Bergmann et al., 2016). It might be useful to administer minimal anesthetic dosages (e.g., of anxiolytics) in the actual experiment to relax mice, which are already habituated. This would create a state of drowsy wakefulness that approximates the “ground truth” against which novel anesthetic protocols could be referenced. Ultimately, multimodal sedation may be instrumental to lock the mouse into a defined spectrum of brain states to minimize variations due to stress, arousal, and distraction, and to prevent effects due to habituation and reward.

### Deep Brain Stimulation, Opto- and Chemogenetics

Functional reorganization and network properties can be further evaluated by using techniques that modulate neural activity from within the brain. The most popular methods are deep brain stimulation, opto- and chemogenetics. These techniques allow one, for example, to evaluate the reorganization of FC in response to locally targeted perturbations. This can be used as a measure for functional complexity of information processing in the anesthetized brain (perturbational complexity index; Casali et al., 2013; Mashour and Hudetz, 2018). Ketamine, for instance, has been shown to preserve cortical functional complexity, which is decreased by propofol with increasing dosage (Sarasso et al., 2015). The signal propagation of locally induced neural activity also provides useful information on FC, which has been shown to be impaired during anesthesia, sleep, and pathological unconsciousness (Massimini et al., 2005; Ferrarelli et al., 2010; Casali et al., 2013). In humans, such investigations are often limited to studying cortical FC using non-invasive transcranial magnetic stimulation. In preclinical fMRI deep brain stimulation can be more easily utilized to induce neural activity in subcortical areas (McIntyre and Anderson, 2016), although the electrodes are prone to cause imaging artifacts, distortions, and local signal loss (Lehto et al., 2018).

Optogenetics employs optical fiber probes that do not directly interfere with functional MR image acquisition. Excitation or inhibition of genetically modified neurons governed by light control can be combined with fMRI to detail spatial and temporal effects on evoked neural activity (Desai et al., 2011; Lai et al., 2015) or FC in remote brain regions (Ryali et al., 2016; Chan et al., 2017). Chemogenetics is a powerful minimally invasive complement to optogenetics, in which neurons are controlled by artificially designed ligands that are injected into the bloodstream (Roth, 2016). Both are important techniques to further investigate the mechanisms of FC (as recently summarized in Mandino et al., 2020), including the impact of distinct transmitter systems in mice. Exclusively selecting, for example, serotonergic neurons of the raphé nuclei (Figure 8D; Grandjean et al., 2019b) or noradrenergic neurons of the locus coeruleus (Zerbi et al., 2019) based on specific gene expression patterns can be used to detail the underpinnings of brain states in anesthesia and the circuits that drive awakening (Carter et al., 2010; Taylor et al., 2016).

## Translational fMRI Across Brain States and Species

The key challenges of preclinical fMRI with a view to translation are: (1) to induce and maintain brain states that correspond with those of calm and relaxed human volunteers; and (2) to assure hemodynamic integrity—in other words, to preserve neurovascular coupling and physiological baseline conditions. Up to this point, we have discussed the general principles of anesthesia and have highlighted the need to recognize and overcome the shortcomings of current anesthetic protocols. This will require strategies to shape, maintain, and unambiguously identify distinct brain states in fMRI that meet the above criteria, to conduct reliable, reproducible studies and translate findings from mice to humans.

### Multimodal Anesthesia in Translational fMRI

Preclinical fMRI aims for an animal that is sufficiently sedated, yet with a fully functional brain. This challenge puts high demands on anesthetic protocols to induce and maintain an intermediate brain state that closely approximates an awake, calm, relaxed, and undistracted state during the fMRI task. Current standard practices involving simple mono-anesthesia are outdated. Balanced multimodal anesthesia, in which two or more drugs are combined to balance their complementary effects, has come into use in the clinic (Brown et al., 2018a) and should be further developed for preclinical studies (see “Sensory Processing and the Key Challenges in Murine fMRI” and “Functional Connectivity and Murine Resting-State fMRI” section; Grandjean et al., 2014; Sharp et al., 2015; Shim et al., 2018).

By reducing the dose of individual compounds, a multimodal protocol reduces excessive stress on a single pathway, taking advantage of complementary modes of action to shape and maintain desired brain states. In principle, respiratory depression or hemodynamic side effects from specific drugs can be attenuated by balancing them with the effects of others. At the same time, combining anesthetic compounds through multimodal anesthesia introduces a higher level of complexity and potential cross-effects that require caution. A rigorous characterization of anesthetic actions will be necessary to understand and adjust the effects of individual drugs in multimodal approaches. This will require that brain states, hemodynamics, and physiology be monitored, evaluated, and reported to the community.

### Defining Brain States in fMRI: Qualifiers and MIND Signature

To properly identify and communicate an anesthetic state in translational fMRI, we first need to define a set of functional characteristics that can identify an induced brain state with high specificity. Their purpose is to evaluate the anesthetic maintenance of an intermediate brain state, and qualify its approximation to the awake state. We will refer to these functional characteristics as *qualifiers*.

Among these qualifiers, we choose one *identifier* that allows the unambiguous assessment of whether a desired intermediate brain state has been maintained during the full length of the fMRI scan, based on a reliable neurophysiological signature pattern. It should permit: (1) identifying a brain state and the integrity of its qualifiers; and (2) comparing this state across subjects, trials, scanners, labs, and species. We will refer to this identifier of a specific brain state as a maintained, intermediate neurophysiologically-determined (MIND) signature.

#### Qualifiers

A number of qualifiers have the potential to illuminate characteristics of brain states and the functional implications that accompany them. The set of such qualifiers is growing rapidly through new findings and the development of novel analytical tools (as summarized in Bonhomme et al., 2019). Here, for the sake of brevity, we list only those that we consider most relevant and sufficient for defining brain states for fMRI in anesthetized animals.

**Figure 9:**
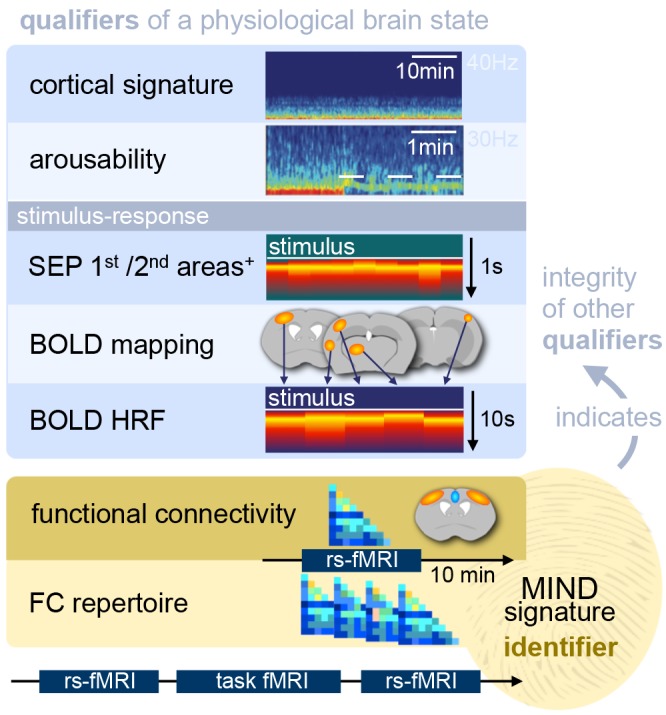
The functional characteristics (qualifiers) and MIND signature (identifier) of a specific brain state induced by an anesthetic protocol. The aim of anesthesia in fMRI is to induce stable sedation, while maintaining a brain state that approximates an awake, calm state. The protocol may be either mono-anesthetic (drug a; dose x) or multimodal (drug a, b, c; dose x, y, z). *Qualifiers* are defined as functional characteristics of an intermediate brain state that has been induced and maintained by the use of anesthesia: The cortical EEG signature permits to monitor anesthetic depth, to evaluate arousability by challenging the stability of a brain state *via* nociception or stress, and to probe stimulus-response features of sensory evoked potentials (SEP). Hemodynamic translation can be evaluated *via* fMRI, whereas shape and magnitude of the hemodynamic response function (HRF) provide information about anesthesia-induced vasomodulation. Anesthetic effects on functional connectivity (FC) across the brain can be assessed based on correlation matrices that depict interrelations of cortical and subcortical areas, including anticorrelations, and the dynamic repertoire of recurring sets of FC patterns over time. FC has great value as an *identifier* of a particular brain state and the integrity of its qualifiers. A fingerprint-like FC signature pattern can be used to unambiguously identify maintained intermediate brain states under anesthesia and communicate them across labs. This signature pattern is termed the *maintained, intermediate neurophysiologically-determined* (MIND) signature. Flanking tasks in fMRI with resting-state scans will permit a researcher to monitor the maintenance or variations in the brain state under anesthesia throughout the fMRI experiment.

Under these conditions, the set of functional characteristics needed to identify an anesthetically induced brain state should include measures of maintenance, arousability, neuronal stimulus–response features, and their hemodynamic translation, as well as FC and its dynamic repertoire (Figure 9). These qualifiers cover a range of informative features to identify and evaluate the degree to which an induced brain state approximates the calm, awake state.

Cortical EEG signatures provide valuable information about cortical synchronization at particular frequency bands (including correlates of phase-amplitude coupling, which can influence cortical information transfer and sensory processing; see “Brain States Under Anesthesia” section; Chamadia et al., 2019), the stability of a maintained intermediate brain state during rest and its response to nociception and stress (see “Metastability of Brain States, Hysteresis, and Behavioral Monitoring” section). Due to its real-time accessibility and high temporal resolution, EEG can be used to assess anesthetic depth and rousability, particularly when aligned with behavioral observations (outside the scanner).

The dynamics of neural stimulus–responses can be directly assessed *via* EEG (whose spatial resolution is limited due to the smoothing properties of the scalp and skull) or invasive electrophysiological recordings of SEP and Ca^2+^ signals in primary, secondary sensory, and higher-order cortical areas. Multichannel recordings (Muller et al., 2018) and large-scale assessment techniques of neural activity, including high-density EEG (Massimini et al., 2005, 2007), Ca^2+^- and voltage-sensitive dye imaging (McVea et al., 2016), can be used to assess the degree of complexity of signal processing and propagation across the cortex at different spatial and temporal scales (see also “Brain States Under Anesthesia” and “Deep Brain Stimulation, Opto- and Chemogenetics” sections). This provides insights into the effects of anesthesia on thalamo-cortical and cortico-cortical sensory transmission, as well as cortical information transfer (see “Brain States Under Anesthesia” section).

Hemodynamic integrity can be evaluated *via* the translation of neural responses into BOLD effects. Comparing SEP or evoked Ca^2+^ signals with BOLD effects reveals whether neural responses are triggered but become lost in hemodynamic translation (Aksenov et al., 2015). Neurovascular coupling varies across cortical areas and has been found to be more reliable in sensory vs. higher-order cortices (Ojemann et al., 2002, 2013; Conner et al., 2011). Therefore, an overall impairment of neurovascular coupling by anesthesia might lead to a selective loss of BOLD effects. Further information about vasoconstrictive or vasodilatory drug effects can be derived from the shape of the hemodynamic response function (HRF; see “Sensory Processing and the Key Challenges in Murine fMRI” section).

Since anesthesia primarily disrupts neural communication across the brain, its effects are most comprehensively reflected by measures of FC. An ideal sedation for fMRI would meet criteria of a close resemblance of the assessed correlation matrices with those of the awake references, including anticorrelated regions, and a rich repertoire of dynamic FC (see “Functional Connectivity and Murine Resting-State fMRI” section).

#### MIND Signature

Another advantage of FC in fMRI is that it can be acquired simply in the form of a resting-state scan without any additional equipment. This makes it a strong candidate measure for determining a MIND signature in translational fMRI. fMRI tasks can be flanked with brief resting-state scans that permit the brain state to be identified at regular points throughout an experiment. Such flanking approaches of FC have previously been shown to reveal valuable information on anesthetic effects on murine fMRI tasks (Nasrallah et al., 2014b; Schroeter et al., 2017; Shim et al., 2018). More advanced FC applications have been used to track spatial and temporal aspects of intermediate brain state transitions as a function of anesthesia in mice (Grandjean et al., 2017a; Gutierrez-Barragan et al., 2018) and rats (Schwalm et al., 2017; Aedo-Jury et al., 2019). Finally, the relations of FC and its structural scaffold have been already used to formulate resting-state dynamics as cortical signatures of anesthesia in monkeys (Barttfeld et al., 2015; Uhrig et al., 2018) and humans (Roberts et al., 2019).

Approaches like these have laid the groundwork for the development of a simple, yet comprehensive signature scheme for the unambiguous identification of anesthetically induced and maintained, intermediate brain states that (1) are adjusted to functionally approximate the awake state and (2) indicate the integrity of a defined set of functional qualifiers. This concept assumes a mechanistic connection between FC and the other qualifiers, so that specific functional properties can be inferred from the detection of a specific MIND signature (Figure 9). Such a mechanistic connection remains to be established—as well as the circumstances in which this assumption can be made with confidence.

### Linking the MIND Signature to Other Qualifiers

Defining a MIND signature that could indicate the cortical state, stimulus–response properties, their hemodynamic translation, and dynamic FC in the form of one unique identifier would be extremely useful. The unambiguous identification of brain states during fMRI would also substantially improve the process of developing (and communicating) novel anesthetic protocols. A verifiable linkage between these qualifiers requires the simultaneous assessment of neural and hemodynamic readouts (see below). However, a large fraction of qualifiers can be assessed in separate measurements. It is reasonable to perform initial anesthetic adjustments outside the MR scanner, based on electrophysiological and behavioral responses to stimuli and cortical signatures during rest (see “Metastability of Brain States, Hysteresis, and Behavioral Monitoring” section), while under a tight control of physiological parameters. It is therefore important to select conditions under which the qualifiers measured outside the scanner can be linked to others obtained during fMRI.

Brain states, and thus stimulus response properties, are likely to change during fMRI scans due to the loud gradient noise, which can trigger activity in subcortical arousal nuclei (see “Metastability of Brain States, Hysteresis, and Behavioral Monitoring” section). In fact, fMRI at high levels of sound pressure substantially boosts BOLD responses to subcutaneous electrostimulation of the rat under α-chloralose, compared to silent fMRI protocols or after deafening by cochleotomy (Burke et al., 2000). Thus, hearing protection is recommended (Reimann et al., 2016). Sound pressure levels depend on the gradient coils, and can be reduced *via* sophisticated design of hardware and pulse sequences (Winkler et al., 2018).

Current efforts are underway to advance silent MR techniques using sweep imaging with Fourier transform (SWIFT; Idiyatullin et al., 2006, 2015). This approach renders obsolete the rapid and pulsed on/off switching of strong magnetic field gradients used for spatial encoding, and substantially lowers the acoustic noise level. SWIFT also permits zero echo time TE, which yields distortion- and artifact-free fMRI, even in the face of severe body motion and with electrodes implanted close to the region of interest (Lehto et al., 2018; Paasonen et al., 2020). This is a major advance for the development of sedation protocols to study FC in freely breathing animals and is further attractive for electrophysiological recordings or deep brain stimulation in fMRI. Until SWIFT is readily available for small-bore animal MR scanners, typical sound levels and vibrations produced by the gradients may be mimicked outside the scanner in the electrophysiological setup to test their effects on cortical states. Simultaneous fMRI and EEG (Sadaghiani et al., 2012; Thompson et al., 2013) or Ca^2+^ imaging (Schwalm et al., 2017; Schlegel et al., 2018) can permit a direct translation between the cortical state and whole-brain hemodynamic responses.

It remains to be clarified to what extent a signature based on FC is sufficient to indicate the integrity of the underlying functional properties of a brain state. Recent studies on the simultaneous imaging of murine cortex-wide FC of hemodynamic and neural calcium fluctuations in the resting state have found a strong relationship between both measurements (Murphy et al., 2017). Both measurements show sufficient resemblance in their intercortical FC for tracking similar brain states under anesthesia (Brier et al., 2019) and similar sets of dynamic FC configurations using a sliding-window approach (Matsui et al., 2018). Transitions in the dynamic FC configurations can be tracked *via* fMRI within a time scale of 1 or 2 s, and a sliding-window length below 1 min (Barttfeld et al., 2015, movie S1; Grandjean et al., 2017a; Lurie et al., 2018), which would be sufficient to resolve effects such as the metastable cortical state transitions that were observed *via* LFP recordings under constant isoflurane concentrations (see “Metastability of Brain States, Hysteresis, and Behavioral Monitoring” sections, Figure 2D; Hudson et al., 2014; Hudson, 2017). However, studies that investigate such brain state transitions under anesthesia linking electrophysiological or calcium recordings with dynamic FC are still pending.

### Limitations in Maintaining Stable Brain States

Even when a brain state is stably maintained over time, it will exhibit a restricted set of recurrent FC configurations that vary within a defined dynamic range (Uhrig et al., 2018). Transitions between two intermediate brain states, as observed for metastable LFP cortical signatures (Hudson et al., 2014), should thus be classifiable as two distinct sets of recurrent FC configurations. Each of these sets varies within the internal dynamic range of its respective brain state. The MIND signature will comprise an indication of the variance of this range (narrow or broad) and the FC patterns that recur (Gutierrez-Barragan et al., 2018). A loss of recurrence and the appearance of new FC configurations would indicate a drift in brain states, and hence a change in anesthetic depth. Transitions in brain states become a confounding factor when they significantly alter the specific functional properties that are relevant for a given fMRI investigation.

The continuous anesthetic infusion *via* intraperitoneal or subcutaneous administration routes may present a practical experimental challenge because the uptake and distribution of agents depends on various biopharmaceutical and biological factors (comprehensively reviewed in Claassen, 1994). Whether and to what extent it is possible to maintain stable brain states under these conditions has to be established according to specific application procedures (Sirmpilatze et al., 2019). Future technical developments and novel methods should produce increasingly detailed functional information that will need to be integrated into single composite fMRI measurements (Breakspear, 2017; Belloy et al., 2018; Roberts et al., 2019; Song et al., 2019). This will produce a MIND signature that identifies brain states with increasing precision.

### The MIND Signature in Translational fMRI

The MIND signature would be valuable as an indicator of specific brain states independent of minor changes in brain anatomy and thus hold even further value for translational fMRI. It would provide a framework for comparing the MIND signatures of humans and mice and facilitate the development of a scheme for systematic evaluation of functional similarity and divergence across species. This would allow for better translation of anesthetic effects from humans to mice and back, and will have three major benefits: (1) anesthetic effects could be referenced against an awake, but calm and relaxed volunteer—even within the same subject; (2) introspective reports could link specific MIND signatures to LOC and to the overall experience; and (3) determining precise translational limitations in general brain functions and specific anesthetic effects, to assess the scope for murine transgenic and optogenetic applications to explore human cognition and consciousness.

### Future Perspectives

The need for effective anesthetic protocols and a better comprehension of their underpinnings is not exclusive to translational fMRI, but is equally important for preclinical researchers studying electrophysiology, Ca^2+^ imaging, and other modalities to probe brain functions. In clinical practice, balanced multimodal anesthesia is common and increasingly being used as a substitute for opioids, especially in light of the current opioid crisis in the US (Brown et al., 2018a). In basic research, cognitive scientists employ anesthesia to probe the neural correlates of consciousness. All these disparate fields produce valuable data that should be taken into account across research domains in pursuit of a deeper understanding of anesthetic effects on animal and human brain functions.

Yet accessing knowledge on anesthetic effects is not always straightforward. Much valuable information is buried in publications that focus on biological research questions and use anesthesia only as an experimental tool. Many anecdotal observations of crucial effects remain unpublished “lab lore” and are not available to the community. Having access to this information could substantially accelerate our progress in detailing anesthetic effects to develop multimodal protocols for a stable sedation with the best possible preservation of brain functions. A community-driven open database of key observations—collected from publications and lab notes—would make this information more accessible, promoting reproducibility and preventing research groups from running into the same “dead ends” already discovered by others. We are currently developing a web-based infrastructure for such an open initiative across neuroimaging communities, to facilitate the exchange of this information. We invite anyone interested in this initiative to contact the corresponding author.

Within the fMRI community, the additional assessments of resting-state fMRI at selected points throughout the experiment would help to specify the actual brain state and make comparisons of data across labs more reliable. The concept of the MIND signature could support this process by defining a set of qualifiers and one identifier readout that permits a reliable indication of a specific brain state and its related functional properties. Our future work is dedicated to further refining and establishing the MIND signature in a collaborative effort.

## Conclusions

To make proper use of translational fMRI for a direct, non-invasive comparison between large-scale brain functions in humans and mice, it is essential to understand the impact of anesthesia on neural physiology. The animal is usually anesthetized to avoid variations due to stress and distraction, with the ultimate aim of achieving stable sedation while maintaining a functional brain state close to that of an awake, resting subject. Maintaining functional brain states and neurovascular coupling are the two major challenges of preclinical fMRI. Most anesthetics affect neural and hemodynamic integrity in different ways, based on their target receptors, which typically assigns them to one of the three main classes of anesthesia. Despite addressing different pathways, all classes exert their effects by spoiling the phase-frequency relationships of remote neuronal populations, which eventually leads to a breakdown of information transfer along the cortical hierarchy and thus LOC. The resulting disruption of higher-order circuits is reflected in FC, although the specifiability of markers for discrete functional stages remain to be clarified. Better knowledge of the mechanisms of action allows to balance different compounds in order to develop multimodal anesthetic protocols for translational fMRI, ideally revealing mainly species-specific variations and not the influence of anesthesia. The concept of the MIND signature has been proposed as an approach to determine and re-identify specific brain states and related functional properties induced by different anesthetic drugs and levels across studies, laboratories, and species.

## Author Contributions

HR: conceiving and drafting the article, agrees to be accountable for all aspects of the work in ensuring that questions related to the accuracy or integrity of any part of the work are appropriately investigated and resolved. TN: revising the article critically, final approval of the submitted version.

## Conflict of Interest

The authors declare that the research was conducted in the absence of any commercial or financial relationships that could be construed as a potential conflict of interest.

## Footnotes

^1^mouse.brain-map.org

^2^bbp.epfl.ch/nexus/cell-atlas

^3^encodeproject.org
